# Modifiable healthcare factors affecting 28-day survival in bloodstream infection: a prospective cohort study

**DOI:** 10.1186/s12879-020-05262-6

**Published:** 2020-07-25

**Authors:** Rebecca N. Evans, Katie Pike, Chris A. Rogers, Rosy Reynolds, Margaret Stoddart, Robin Howe, Mark Wilcox, Peter Wilson, F. Kate Gould, Alasdair MacGowan

**Affiliations:** 1 Clinical Trials and Evaluation Unit, Bristol Trials Centre, Bristol Medical School, University of Bristol, Level 7, Bristol Royal Infirmary, Queen’s Building, Bristol, BS2 8HW UK; 2 Population Health Sciences, Bristol Medical School, University of Bristol, Bristol, UK; 3 Infection Sciences, Pathology, North Bristol NHS Trust, Bristol, UK; 4 Public Health Wales, Microbiology, Cardiff University Hospital of Wales, Cardiff, UK; 5 Department of Microbiology, Leeds General Infirmary, Leeds Teaching Hospitals NHS Trust, Leeds, UK; 6 Clinical Microbiology, UCLH NHS Foundation Trust, London, UK; 7 Department of Medical Microbiology, Freeman Hospital, Newcastle-upon-Tyne NHS Trust, Newcastle-Upon-Tyne, UK

**Keywords:** Bloodstream infection, Mortality, Observational cohort, Modifiable, Appropriate antimicrobial therapy

## Abstract

**Background:**

Bloodstream infection is common in the UK and has significant mortality depending on the pathogen involved, site of infection and other patient factors. Healthcare staffing and ward activity may also impact on outcomes in a range of conditions, however there is little specific National Health Service (NHS) data on the impact for patients with bloodstream infection. *Bloodstream Infections – Focus on Outcomes* is a multicentre cohort study with the primary aim of identifying modifiable risk factors for 28-day mortality in patients with bloodstream infection due to one of six key pathogens.

**Methods:**

Adults under the care of five NHS Trusts in England and Wales between November 2010 and May 2012 were included. Multivariable Cox regression was used to quantify the association between modifiable risk factors, including staffing levels and timing of appropriate therapy, and 28-day mortality, after adjusting for non-modifiable risk factors such as patient demographics and long-term comorbidities.

**Results:**

A total of 1676 patients were included in the analysis population. Overall, 348/1676 (20.8%) died within 28 days. Modifiable factors associated with 28-day mortality were ward speciality, ward activity (admissions and discharges), movement within ward speciality, movement from critical care, and time to receipt of appropriate antimicrobial therapy in the first 7 days. For each additional admission or discharge per 10 beds, the hazard increased by 4% (95% CI 1 to 6%) in medical wards and 11% (95% CI 4 to 19%) in critical care. Patients who had moved wards within speciality or who had moved out of a critical care ward had a reduction in hazard of mortality. In the first 7 days, hazard of death increased with increasing time to receipt of appropriate antimicrobial therapy.

**Conclusion:**

This study underlines the importance of appropriate antimicrobials within the first 7 days, and the potential for ward activity and ward movements to impact on survival in bloodstream infection.

## Key points

Modifiable risk factors associated with 28-day mortality after BSI are ward speciality, ward activity, ward movement within speciality, movement from critical care and time to receipt of appropriate antibiotics in the first 7 days.

## Background

At least 100,000 patients have an episode of bloodstream infection (BSI) each year in England, Wales and Northern Ireland [1]. Depending on the pathogen involved, underlying patient characteristics, the severity of infection and treatment provided the death rate from these infections can reach 15–25% at 30 days and 50% at 3 years [2–4]. Poor outcomes are known to be related to several non-modifiable patient factors including age, comorbidities, severe sepsis, source of infection, neutropenia, type of infection, and intensive care (ICU) admission [2, 5–10]. In contrast, timely appropriate antimicrobial chemotherapy and removal of infected prosthetic materials have been found to be beneficial – but data are typically from single-centre studies, and information on the estimated size of these effects is limited. Staffing levels are known to impact a range of care quality measures, including patient mortality [11–13]. In addition, nursing skill mix and the use of non-permanent staff may increase the rates of hospital-acquired infection [14]. However there is less information on the impact of staffing on infection outcomes in general, and none on its impact on BSI outcomes in particular [14, 15].

The Bloodstream Infections – Focus on Outcomes (BSI-FOO) study is a prospective cohort study designed to quantify modifiable risk factors for death (from all causes) within 28 days of onset of BSI caused by six key pathogens: 1) methicillin-resistant *Staphyloccos aureus* (MRSA); 2) methicillin-susceptible *S. aureus* (MSSA); 3) non-Extended-spectrum beta-lactamase (ESBL)-producing *Escherichi coli*; 4) any ESBL-producing member of the family Enterobacteriales; 5) *Pseudomonas aeruginosa*; 6) any species of *Candida.*

## Methods

### Study design

BSI-FOO is a prospective multicentre cohort study conducted across five NHS acute hospital trusts in England and Wales. The study period was from November 2010 to May 2012, but for administrative reasons the study opened and closed on different dates in the five centres. Data were collected from routine care, investigations or tests and recorded according to usual clinical practice. The National Information Governance Board approved the use of such routinely collected data without individual patient consent under section 251 of the NHS Act 2006. Patients were followed up for 28 days from the date the diagnostic blood sample was taken.

### Study population

Adults (≥18 years old) receiving in-patient NHS hospital care and having a clinically significant BSI with an organism in one or more of the six key pathogen groups were included. Non-ESBL-producing *E. coli* is one of the most prevalent pathogen of BSI in the UK, so to ensure all pathogen groups were adequately represented, a random sample of one-third of episodes caused by non-ESBL-producing *E. coli* and all episodes caused by the other six key pathogens were included. The number of cases at each of the hospitals during the study period determined the sample size.

Patients with HIV-positive serology, cystic fibrosis, on an end-of-life care pathway when the blood sample was taken, in the custody of HM Prison Service of England or Wales, not receiving NHS care, not an in-patient when the blood sample was taken and not admitted shortly afterwards, or discharged on the day the sample was taken, with notes irretrievably missing or a generalised refusal to take part in research noted in medical records were excluded. Duplicate blood cultures i.e. samples from the same infection episode, were also excluded.

### Outcome measures

The primary outcome was time to death up to 28 days of taking the diagnostic positive blood sample. Death within seven days was a secondary outcome. Other outcomes, to be reported elsewhere, were time to resolution of fever, and length of hospital stay.

### Risk factors

An episode of infection began when the first positive blood sample confirming BSI was taken (defined as day/time 0). Any factors present before time 0 were considered non-modifiable (see Supplemental Table 1, Additional file 1). Modifiable risk factors considered were aspects of hospital care received from time 0 onwards, which included staffing levels, ward activity (number of admissions/discharges), movements between wards, timing of appropriate therapy and continuing presence of intravenous lines and catheters (see Supplemental Table 2, Additional file 1). Within each centre, ward level information including ward speciality, staffing and activity was collected from day 0 to 7, for the ward where the patient spent most of their day. Overall staffing levels, including healthcare assistants, trust-employed nurses and agency nurses, was averaged across three shifts (early, late and night) and defined as staff:bed ratio (number of staff per bed). Ward activity per ped was defined similarly. The presence or absence of central lines, peripheral lines and urinary catheters was observed on day 0, and their presence on days 1 to 28 determined using the date of removal.

Antimicrobial therapy was defined as ‘appropriate’ if the organism was susceptible to the antimicrobial prescribed, and therapy continued for at least 36 h to allow therapeutic effect. Susceptibility was judged first by test results for the antimicrobial prescribed (if available), then by inference from test results for related antimicrobials. In the absence of any relevant test results, susceptibility was deduced from the inherent susceptibility/resistance profile for the species concerned [16]. If treatment was changed from one appropriate antimicrobial to another, this was treated as a single period of appropriate therapy providing that the next therapy began within 24 h of the last dose of the previous therapy.

### Statistical analyses

The analysis population consisted of all eligible participants entered into the study. Repeat episodes (distinct infection episodes within the same patient) and patients with polymicrobial episodes (infections involving more than one microbial species) were excluded. The statistical model was built in two stages:

#### Stage one model

The non-modifiable risk factors were assessed univariately and factors associated with mortality were identified using Cox regression analysis with a 20% significance level. These identified factors were then considered for inclusion in a multivariable model with factors identified using backwards selection. The estimated ln(hazard ratio) for each factor included in the model were then used to derive a “risk score” for each patient. The proportional hazards assumption was assessed and if the assumption was not met then the model was stratified by the variable(s) causing non-proportional hazards. Multivariable fractional polynomial models were used to select the best-fitting functional form for continuous variables.

#### Stage two model

All modifiable risk factors were included in the model regardless of statistical significance, with organism and the risk score derived in stage one included as covariates. Episodes were split at daily intervals from day 0 to 28 with ward speciality, central line, peripheral line, urinary catheter, ward movements, staffing levels, ward activity, and antimicrobial therapy variable values updated at each interval (see Supplemental Table 2, Additional file 1). Ward data was only collected up to day 7 and so for patients who survived and were discharged after day seven, ward speciality, staffing, ward activity and ward movements were assumed be constant for the remaining days (up to death/discharge or day 28). For risk factors which were bounded by survival time e.g. time to receipt of appropriate therapy and number of ward movements, time-dependent variables were used within the data framework. The value of the time-dependent exposure was calculated as a cumulative count of ward movements up until that day. So, for each day at risk, the ward movement count was increased by one if the patient moved wards or remained the same if the patient did not. Similarly, a cumulative count of days before first receipt of appropriate antimicrobial therapy was used. That is, on day 0 time to receipt of appropriate therapy was 0 for all patients, remaining at 0 each day for patients who received appropriate therapy on day 0 otherwise increasing by one for each additional day until receipt of first appropriate therapy. This ensured that on day 3, for example, the maximum time to appropriate therapy was 3 days, for both survivors and deceased.

Interactions between ward speciality and ward activity and between ward speciality and staffing levels were included in the model regardless of statistical significance. The following interaction terms were then considered for potential inclusion: organism with each of risk score, central line, peripheral line, urinary catheter and time to appropriate antimicrobial therapy, and ward speciality with within-ward speciality movements. A forward stepwise approach was taken to select interactions to be included in the final model, using likelihood ratio tests to compare nested models with a 10% significance level. This level was chosen to increase statistical power whilst ensuring that not the number of events per variable did not exceed 10 [17].

Model fit was assessed using standard methods and calibration was assessed by comparing observed event rate for patients in each decile of predicted event rates. Collinearity was examined using the variance inflation factor with values < 5 considered acceptable. The proportional hazards assumption was assessed as for the stage one model. If non-proportional hazards were indicated, then time was categorised into periods where proportional hazards appeared valid and an interaction between this categorised time and the variable with non-proportional hazards was added to the model.

The risk score derived from the stage one model was also used for the analysis for the secondary outcome, so only stage two was repeated for 7-day mortality.

Missing data was assumed to be missing at random and missing values were imputed using multiple imputation (45 imputations) and the results were combined using Rubin’s rules. Non-normally distributed variables were transformed prior to imputation. If a suitable transformation could not be found or the imputation procedure imputed values outside valid ranges then predictive mean matching was used for the imputation of that variable. All variables that were in the primary analysis model, potential auxiliary variables (Supplemental Table 1, Additional file 1), indicator for death and the log of survival time were included in the imputation procedure. We intended to allow for any interaction terms in the main analysis model in the imputation procedure by imputing separately for each category of one of the variables involved in any interactions. However, unfortunately, the imputation procedure would not converge, and it was not possible to include the interactions in the imputation. The model selection process was performed on a single imputed dataset so that log-likelihood statistics could be calculated and compared. All analyses were performed in Stata version 14.0 (StataCorp LP, College Station, Texas, USA).

#### Sensitivity analysis

In the main analysis, an antimicrobial treatment was considered ‘appropriate’ if the organism was susceptible to the antimicrobial prescribed and the therapy continued for at least 36 h; and was not considered appropriate if the patient died within 36 h of starting treatment. However, this may lead to inflated effect estimates as the death could be viewed as a consequence of not receiving the therapy. A sensitivity analysis with the “36-h rule” removed was performed to assess this possibility and we also investigated a 12-h and 24-h rule. A complete case analysis was also performed to assess the impact of the multiple imputation.

## Results

In total, 1828 patients (1903 eligible blood samples) were recruited; 227 repeat and/or polymicrobial episodes were excluded leaving an analysis population of 1676 patients: 116 with *Candida*, 168 ESBL-producing Enterobacteriales, 542 *E. coli*, 237 *P. aeruginosa*, 513 MSSA and 100 MRSA (Supplemental Figure 1, Additional file 1).

**Fig. 1:**
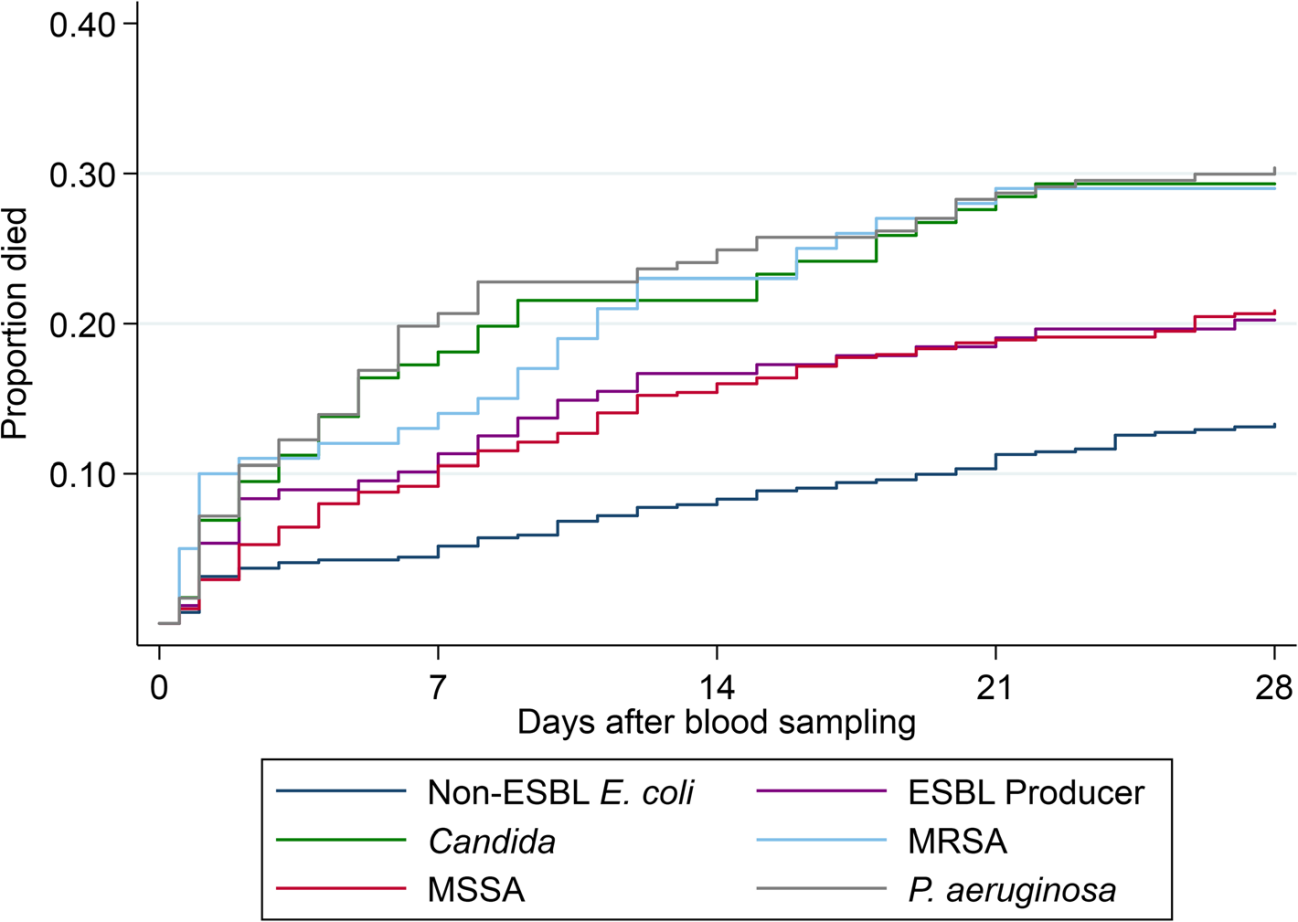
Kaplan-Meier curve for 28-day survival, by organism. Abbreviations: ESBL = Extended-spectrum beta-lactamase, MRSA- Methicillin-resistant *S. aureus*, MSSA = Methicillin-susceptible *S. aureus*

Patients with *P. aeruginosa* had the highest death rate within 28 days (30.4%; 95% confidence interval (CI) 24.6 to 36.7%) and non-ESBL-producing *E. coli* the lowest (13.3%; 95% CI 10.5 to 16.4%). Patients with MSSA and ESBL producers experienced similar death rates (20.9%; 95% CI 17.4 to 24.6% and 20.2%; 95% CI 14.4 to 27.1% respectively), as did patients with MRSA and *Candida* (29.0%; 95% CI 20.4 to 38.9% and 29.3%; 95% CI 21.2 to 38.5%). A Kaplan-Meier curve for the unadjusted survival rates is given in Fig. 1.

Non-modifiable factors including demography and patient comorbidities are summarised by 28-day survival status in Table 1 and Supplemental Table 3, Additional file 1. Approximately 55% of the patients were male, with a median age of 68.5 years (interquartile range (IQR) 53.0 to 80.0).

**Table 1:** Summary table of non-modifiable risk factors, by 28-day survival status

Risk factor		Survived (***n*** = 1328)		Died (***n*** = 348)		Overall (***n*** = 1676)	
		n	%	n	%	n	%
**Patient measures**							
Age	Median (IQR)	67.0	(51.0, 79.0)	74.0	(62.0, 83.5)	68.5	(53.0, 80.0)
Male		728/1328	54.8%	191/348	54.9%	919/1676	54.8%
Body mass index ^a^	Mean (SD)	26.1	(6.7)	25.3	(5.9)	26.0	(6.6)
**Patient medical history**							
Chemotherapy in month before date 0		201/1328	15.1%	57/348	16.4%	258/1676	15.4%
Any tumour within last 5 years		419/1328	31.6%	156/348	44.8%	575/1676	34.3%
Surgery (overnight stay) ≤7 days before date 0		118/1327	8.9%	34/348	9.8%	152/1675	9.1%
Burn requiring admission ≤7 days before date 0		3/1326	0.2%	1/347	0.3%	4/1673	0.2%
Cardiac arrest ≤7 days before date 0		5/1328	0.4%	5/348	1.4%	10/1676	0.6%
Renal support ≤7 days before date 0		81/1328	6.1%	44/348	12.6%	125/1676	7.5%
Myocardial infarction ≤7 days before date 0		128/1328	9.6%	44/348	12.6%	172/1676	10.3%
**Infection severity measures**							
Mental Disorientation:							
None		1113/1327	83.9%	257/348	73.9%	1370/1675	81.8%
Grade I		66/1327	5.0%	20/348	5.7%	86/1675	5.1%
Grade II		86/1327	6.5%	42/348	12.1%	128/1675	7.6%
Grade III		54/1327	4.1%	20/348	5.7%	74/1675	4.4%
Grade IV		8/1327	0.6%	9/348	2.6%	17/1675	1.0%
Temperature (°C) at time 0 ^b^	Mean (SD)	38.2	(1.0)	37.7	(1.2)	38.1	(1.1)
eGFR (mL/min/1.73m^2^) ^c^	Median (IQR)	65.0	(37.0, 90.0)	52.5	(26.5, 84.0)	62.0	(35.0, 90.0)
Serum albumin (g/L) ^d^	Mean (SD)	32.6	(7.5)	27.2	(7.9)	31.5	(7.9)
Bilirubin total (micromol /L) ^e^	Median (IQR)	12.0	(7.0, 20.5)	13.0	(8.0, 23.0)	12.0	(8.0, 21.0)
Neutrophil count at day 0 or closest (× 10^9^/L) ^f^	Median (IQR)	9.3	(5.4, 13.8)	10.2	(4.8, 15.3)	9.5	(5.3, 14.1)
Systolic BP at day 0 or closest (mmHg) ^g^	Mean (SD)	122.9	(26.2)	117.9	(28.7)	121.9	(26.8)
On intravenous fluids at day 0		450/1324	34.0%	165/348	47.4%	615/1672	36.8%
On ventilation at day 0		90/1323	6.8%	66/348	19.0%	156/1671	9.3%
On vasopressor drugs at day 0		60/1327	4.5%	48/348	13.8%	108/1675	6.4%
Systemic corticosteroids in last 24 h		149/1324	11.3%	81/347	23.3%	230/1671	13.8%
Early warning score nearest to day 0							
≤ 3		468/687	68.1%	99/173	57.2%	567/860	65.9%
> 3		219/687	31.9%	74/173	42.8%	293/860	34.1%
**Patient comorbidities at date 0**							
Congestive heart failure		151/1328	11.4%	61/348	17.5%	212/1676	12.6%
Peripheral vascular disease		103/1328	7.8%	43/348	12.4%	146/1676	8.7%
Cerebrovascular disease		198/1328	14.9%	74/348	21.3%	272/1676	16.2%
Hemiplegia		50/1328	3.8%	18/348	5.2%	68/1676	4.1%
Dementia		99/1327	7.5%	39/348	11.2%	138/1675	8.2%
Chronic obstructive pulmonary disease		160/1327	12.1%	57/348	16.4%	217/1675	13.0%
Connective tissue disease		117/1328	8.8%	30/348	8.6%	147/1676	8.8%
Peptic ulcer disease		86/1328	6.5%	31/348	8.9%	117/1676	7.0%
Ascites		48/1328	3.6%	32/348	9.2%	80/1676	4.8%
Diabetes		276/1328	20.8%	81/348	23.3%	357/1676	21.3%
Modified Child-Pugh score ^h^	Median (IQR)	6.0	(6.0, 7.0)	7.0	(6.0, 8.0)	7.0	(6.0, 8.0)
Modified Charlson score ^i^	Median (IQR)	3.0	(2.0, 4.0)	4.0	(2.0, 5.0)	3.0	(2.0, 4.0)
Infected foreign body at time 0		16/1327	1.2%	3/347	0.9%	19/1674	1.1%
**Source of infection (CDC criteria)**							
Bone and joint		59/1327	4.4%	6/348	1.7%	65/1675	3.9%
Cardiovascular system		25/1327	1.9%	5/348	1.4%	30/1675	1.8%
Central nervous system		9/1327	0.7%	0/348	0.0%	9/1675	0.5%
Eye, ear, nose, throat or mouth		3/1327	0.2%	1/348	0.3%	4/1675	0.2%
Gastrointestinal system		134/1327	10.1%	16/348	4.6%	150/1675	9.0%
Line infection – central venous line		123/1327	9.3%	15/348	4.3%	138/1675	8.2%
Line infection – peripheral venous line		20/1327	1.5%	7/348	2.0%	27/1675	1.6%
Lower respiratory tract		61/1327	4.6%	57/348	16.4%	118/1675	7.0%
Reproductive tract		9/1327	0.7%	2/348	0.6%	11/1675	0.7%
Skin and soft tissue		98/1327	7.4%	20/348	5.7%	118/1675	7.0%
Surgical site infection		37/1327	2.8%	4/348	1.1%	41/1675	2.4%
Urinary tract infection		386/1327	29.1%	61/348	17.5%	447/1675	26.7%
Site uncertain		363/1327	27.4%	15,446/348	44.3%	517/1675	30.0%
**Organisational factors**							
Admission from nursing home		97/1327	7.3%	40/348	11.5%	137/1675	8.2%
Length of prior hospital stay (days)	Median (IQR)	1.0	(0.0, 10.0)	5.0	(0.0, 14.0)	1.0	(0.0, 11.0)
Hospital or community acquired infection							
Hospital		548/1328	41.3%	201/348	57.8%	749/1676	44.7%
Community		780/1328	58.7%	147/348	42.2%	927/1676	55.3%
Speciality of consultant on day 0:							
Medicine		559/1217	45.9%	171/333	51.4%	730/1550	47.1%
High dependency medicine		202/1217	16.6%	80/333	24.0%	282/1550	18.2%
Major surgery		355/1217	29.2%	60/333	18.0%	415/1550	26.8%
Minor surgery		9/1217	0.7%	3/333	0.9%	12/1550	0.8%
Other		92/1217	7.6%	19/333	5.7%	111/1550	7.2%

Abbreviations: SD Standard deviation, IQR Interquartile range, eGFR Estimated glomerular filtration rate, BP Blood pressure, CDC Centres for Disease Control and Prevention

^a^ Data missing for 799 patients (604 survived, 195 died)

^b^ Data missing for 30 patients (20 survived, 10 died)

^c^ Data missing for 118 patients (98 survived, 20 died)

^d^ Data missing for 200 patients (161 survived, 39 died)

^e^ Data missing for 267 patients (216 survived, 51 died)

^f^ Data missing for 139 patients (110 survived, 29 died)

^g^ Data missing for 246 patients (196 survived, 50 died)

^h^ Data missing for 1075 patients (867 survived, 208 died)

^i^ Data missing for 377 patients (299 survived, 78 died)

Modifiable risk factors and the presence of lines are summarised by 28-day survival status in Table 2 and Supplemental Table 4, Additional file 1. Approximately two-thirds of patients had a line present at time 0 and both central and peripheral lines at time 0 were more common in patients who died (30.5% vs 22.6, and 62.5% vs 46.8%, respectively). The average number of nurses per 10 beds on day 0 was slightly higher for patients who died compared to those who survived (median 1.7 nurses [IQR 1.2 to 7.0] vs 1.4 [IQR 1.1 to 2.1]). Ward activity on day 0 was similar with a median of 3.7 admissions/discharges per 10 beds (IQR 1.9 to 7.3) for patients who survived and 3.5 admissions/discharges per 10 beds (IQR 1.8 to 5.5) for patients who died. Overall, 84.5% of patients (1416/1676) received appropriate antimicrobial therapy with a median time to receipt of 7 h (IQR 1–40) (Supplemental Table 5, Additional file 1).

**Table 2:** Ward speciality, ward movements, staffing and ward activity, by 28-day survival status

Ward variable		Survived (***n*** = 1328)		Died (***n*** = 348)		Overall (***n*** = 1676)	
		n	%	n	%	n	%
**Ward speciality on day 0**							
Medicine		738/1316	56.1%	186/345	53.9%	924/1661	55.6%
Critical care		138/1316	10.5%	96/345	27.8%	234/1661	14.1%
Major surgery		355/1316	27.0%	50/345	14.5%	405/1661	24.4%
Minor surgery		22/1316	1.7%	3/345	0.9%	25/1661	1.5%
Other		63/1316	4.8%	10/345	2.9%	73/1661	4.4%
**Type of ward movements between day 0 and 7**							
Movement to critical care		87/1301	6.7%	30/337	8.9%	117/1638	7.1%
Movement from critical care		98/1301	7.5%	8/337	2.4%	106/1638	6.5%
Movement within a ward speciality		347/1301	26.7%	48/337	14.2%	395/1638	24.1%
Movement from medicine to surgery		82/1301	6.3%	7/337	2.1%	89/1638	5.4%
Movement from surgery to medicine		30/1301	2.3%	5/337	1.5%	35/1638	2.1%
**Staffing and ward activity on day 0**							
Average number of nurses per 10 beds ^a^	Median (IQR)	1.4	(1.1, 2.1)	1.7	(1.2, 7.0)	1.4	(1.1, 2.4)
Average number of HCA per 10 beds ^b^	Median (IQR)	0.6	(0.4, 0.8)	0.7	(0.4, 0.9)	0.6	(0.4, 0.8)
Average number of agency staff per 10 beds ^c^	Median (IQR)	0.0	(0.0, 0.3)	0.1	(0.0, 0.3)	0.0	(0.0, 0.3)
Average number of total staff per 10 beds ^d^	Median (IQR)	2.3	(1.9, 2.8)	2.6	(2.0, 7.4)	2.3	(1.9, 3.1)
Ward activity per 10 beds ^e f^	Median (IQR)	3.7	(1.9, 7.3)	3.5	(1.8, 5.5)	3.7	(1.9, 6.9)

Abbreviations: HCA Healthcare assistant

^a^ Data missing for 276 patients (234 survived, 42 died)

^b^ Data missing for 277 patients (235 survived, 42 died)

^c^ Data missing for 288 patients (245 survived, 43 died)

^d^ Data missing for 300 patients (255 survived, 45 died)

^e^ Data missing for 153 patients (133 survived, 20 died)

^f^ Ward activity is defined as the number of patients admitted + number of patients discharged

There was some suggestion of slightly reduced numbers of nurses at weekends compared to weekdays, but the average number of health care assistants (HCA) and agency staff did not appear to change throughout the course of a week (Supplemental Figure 2, Additional file 1). Ward activity followed a similar trend to that of nursing levels, the quietest days being Saturday and Sunday with a median of 1.8 (IQR 1.5 to 4.5) and 1.5 (IQR 0.7 to 2.9) admissions/discharges per 10 beds, respectively, compared to a median above 3 on all other days (Supplemental Figure 3, Additional file 1).

**Fig. 2:**
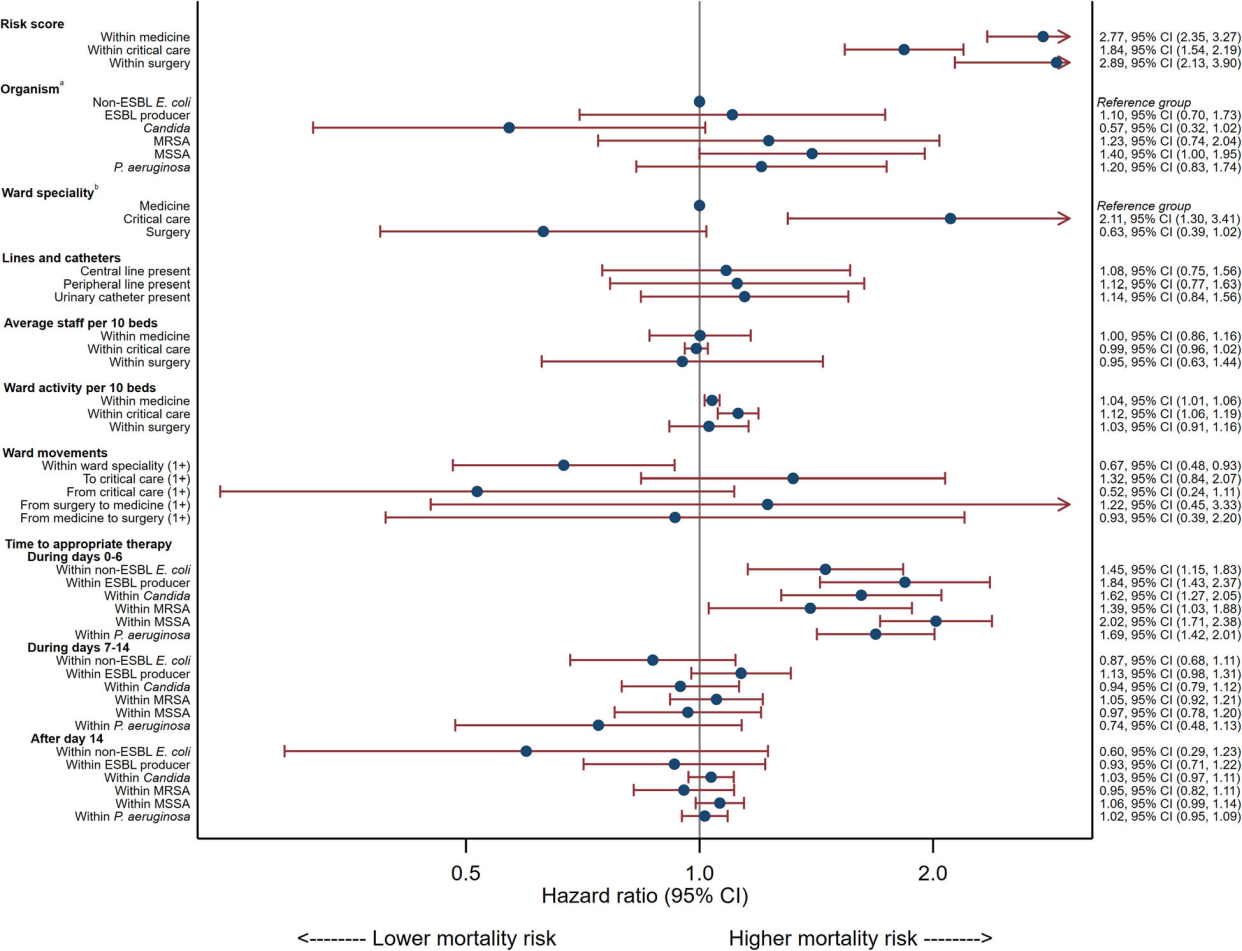
Adjusted Cox model of modifiable risk factors on 28-day mortality. **a** Effect of organism is given for the time period 0 to 6 days, when time to appropriate therapy is 1 day. **b** Effect of ward speciality is given for the median number of staff per 10 beds, median ward activity and median risk score. Abbreviations: ESBL = Extended-spectrum beta-lactamase, MRSA- Methicillin-resistant *S. aureus*, MSSA = Methicillin-susceptible *S. aureus*, CI=Confidence interval

All patients were followed up for the full 28-day follow-up period and were therefore not censored at hospital discharge. The model used to derive the risk score from non-modifiable risk factors for mortality is shown in Supplemental Table 6, Additional file 1. The model for modifiable risk factors, after adjusting for risk score and organism is detailed in Fig. 2 and Supplemental Table 7, Additional file 1. Interaction terms that were included in the model (*p* < 0.1) were i) risk score by ward speciality, ii) time to receipt of appropriate antimicrobial therapy by organism. Model checks suggested that the hazards for time to receipt of appropriate antimicrobial therapy were not proportional. Therefore, follow-up time was categorised into three intervals: days 0–6, days 7–13, and day 14 onwards, and the effect of time to appropriate antimicrobial therapy was estimated separately for each interval.

The final model indicated that ward speciality (ICU), increased ward activity, and time to receipt of appropriate antimicrobial therapy in the first 7 days were associated with increased hazard of death and ward speciality (surgical), movement within ward speciality and movement from a critical care ward were associated with decreased hazard of death within 28 days, after adjustment for risk score and all other factors included in the model (Fig. 2).

The effect of risk score on mortality was greatest for patients in surgical (Hazard ratio (HR) 2.89; 95% CI 2.13 to 3.90) and medical wards (HR 2.77; 95% CI 2.35 to 3.27). For patients in critical care, the effect was still highly statistically significant, but with a smaller effect size (HR 1.84; 95% CI 1.54 to 2.19). The presence of central lines, peripheral lines and urinary catheters were not significantly associated with 28-day mortality.

Patients in critical care wards had a 111% increase (95% CI 30 to 241%) in hazard of mortality within 28 days and patients in surgical wards a 37% decrease (95% CI 2% increase to 61% decrease), compared to patients in a medical ward. These values are estimated at the median staffing, ward activity and risk score values, due to interactions between ward speciality and these terms.

The average staff per 10 beds was not significantly associated with 28-day mortality, although the estimated effect was greater for surgical wards (HR 0.95; 95% CI 0.63 to 1.44) compared to medicine (HR 1.00; 95% CI 0.86 to 1.16) and critical care (HR 0.99; 95% CI 0.96 to 1.02). In terms of ward activity, in a medical ward for each increase in 1 admission or discharge per 10 beds there was a 4% (95% CI + 1% to + 6%) increased hazard of death within 28 days. This increase in hazard was slightly higher in a critical care ward (12%; 95% CI + 6% to + 19%) and negligible in a surgical ward (3%; 95% CI − 9% to + 16%).

Patients who had moved wards within a speciality had a 33% reduced (95% CI: − 7% to − 52%) hazard of death within 28 days. There was also a 48% reduction (95% CI − 76% to + 11%) for patients who had moved out of a critical care ward. There was no evidence to suggest movement to a critical care ward, movement from surgery to medicine or from medicine to surgery impacted on 28-day mortality.

The effect of time to receipt of appropriate antimicrobial therapy varied depending on organism and time. There was a highly significant effect for all organisms for each day delay during the first week. The effect was greatest for MSSA with a 102% increase (95% CI + 71% to + 138%) in hazard of mortality associated with each day delay until the receipt of first appropriate antimicrobial therapy, and lowest for MRSA, with a corresponding 39% increase (95% CI + 3% to + 88%) in hazard of mortality. For patients who survived to day 7, the effect of time to receipt of first appropriate therapy on 28-day mortality was not statistically significant after day 7. Predicted and observed risks are given by deciles of predicted risk for 28-day mortality in Supplemental Table 8, Additional file 1.

The sensitivity analyses assessing the impact of changing the “36-h rule” in defining appropriate antimicrobial therapy are given in Supplemental Figures 4, 5 and 6, Additional file 1. The sensitivity analyses showed similar effects, suggesting that the chosen definition is sensible. The complete case analysis also gave similar results (Supplemental Figure 7, Additional file 1).

**Fig. 3:**
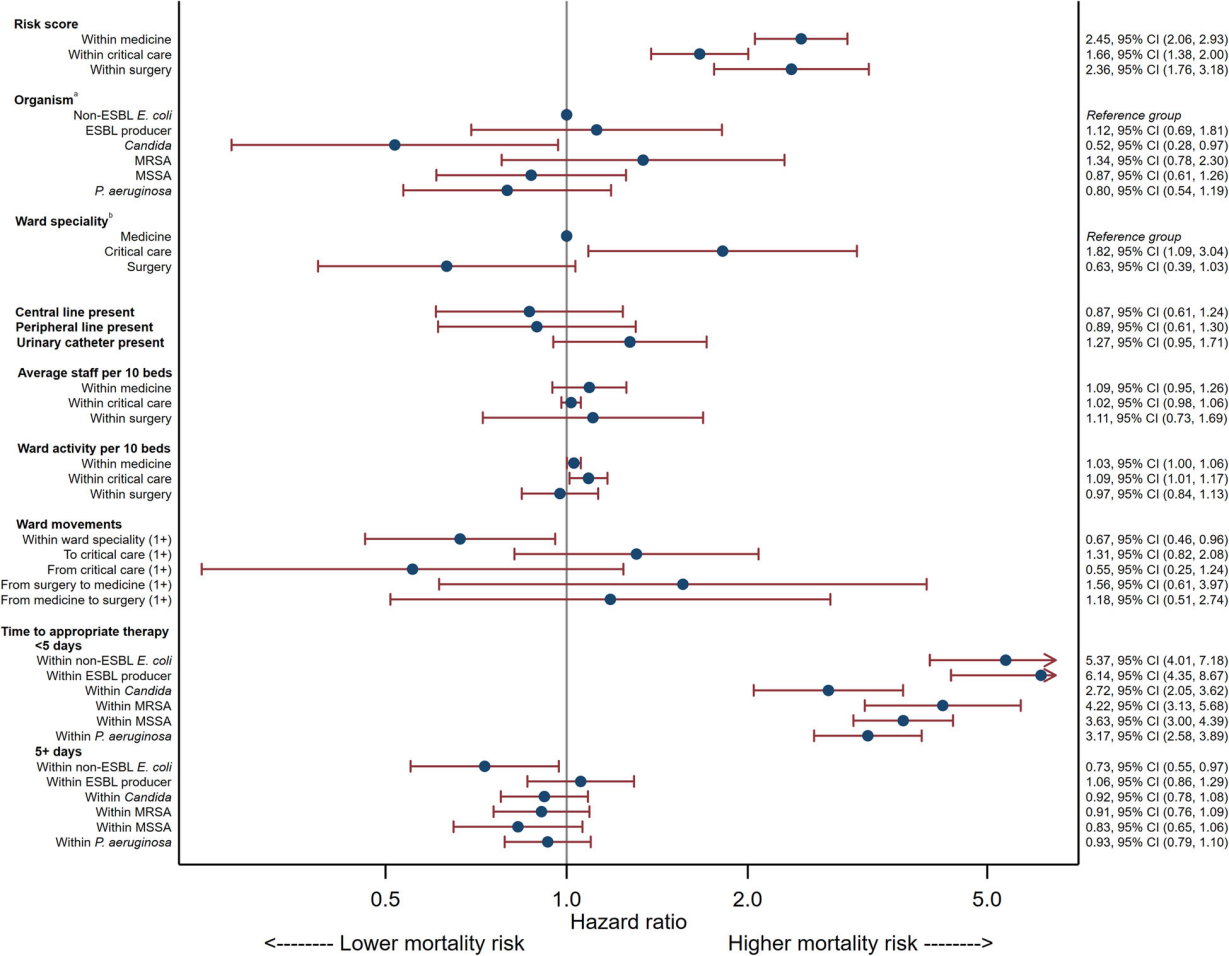
Adjusted Cox model of modifiable risk factors on 7-day mortality. **a** Effect of organism is given for the time period 0 to 5 days and when time to appropriate therapy is 1 day. **b** Effect of ward speciality is given for the median number of staff per 10 beds, median ward activity and median risk score. Abbreviations: ESBL = Extended-spectrum beta-lactamase, MRSA- Methicillin-resistant *S. aureus*, MSSA = Methicillin-susceptible *S. aureus*, CI=Confidence interval

Of the 348 deaths within 28 days, just over half occurred within the first 7 days (Supplemental Table 9, Additional file 1). Across the different organisms 7-day mortality followed a similar pattern to 28-day mortality, patients with *P. aeruginosa* having the highest death rate (20.7%) and non-ESBL-producing *E. coli* the lowest (5.2%). The analyses indicated that ward speciality, ward activity, movements within ward specialities, and time to receipt of appropriate antimicrobial therapy in the first 5 days were all risk factors associated with mortality within 7 days, after adjustment for other factors (Fig. 3 and Supplemental Table 10, Additional file 1).

## Discussion

This report describes modifiable risk factors for 28-day mortality in patients with bloodstream infection due to one of six key pathogens. Our main findings were that:

- For all the pathogen groups studied timely appropriate antimicrobial therapy was associated with improved mortality over 28 days, and the effect of each day delay was most marked in the first 7 days.
- Increased ward activity (admissions and discharges) was associated with increased hazard of death within medical wards and especially in critical care wards.

The analysis of risk factors for mortality was split into those classified as non-modifiable factors and those that could be modified by changes in organisation or patient management. For the pathogen groups studied, ward speciality, ward activity, ward movement within speciality, movement from critical care and time to receipt of appropriate antibiotics were all independently associated with mortality.

The reduced hazard of death within 28 days for patients who moved wards within ward specialty or had moved out of a critical care ward could be a result of healthier patients being more likely to be moved or a result of improving condition. Similarly, the association between ward specialty and mortality is likely to be related to the severity and complexity of patients admitted to the ward.

There are a large number of publications relating organisational and management factors to infection control performance in acute hospitals including workforce and workload [18]. However, there are no previous studies relating ward staffing and activity to infection outcomes. In our descriptive analysis of modifiable factors, patients who died were on wards where the average number of nurses per 10 beds on day 0 was slightly higher than for those who survived, but this may reflect the higher mortality in intensive care units where nursing staffing was much higher. After adjustment for other factors there was no evidence of a statistically significant effect of staffing levels on 28-day mortality. Interestingly, the number of NHS-employed nurses, healthcare assistants or agency staff working did not vary greatly by day of week, although nurse numbers were slightly lower at the weekend. However, ward activity was markedly lower at weekends – which is perhaps not surprising despite the current drive in the NHS towards a 7 day working week [19]. Ward activity was highest on the day blood samples were taken and diminished over the following week, possibly as patients are moved from high activity settings such as emergency departments or admissions units to wards having more stable populations of patients undergoing recovery. It has been shown that increasing exposure to shifts with high turnover of patients is associated with an increase in the risk of death, however there is less information on the impact of workload on infection outcomes in particular [20]. In an adjusted model where ward activity was updated daily to reflect the ward activity where the patient spent most of the day, increased ward activity was associated with an increased hazard of death within 28 days.

Appropriate antimicrobial therapy has been shown to reduce mortality based on a large number of publications over the last 20 years [21]. There are several more recent systematic reviews and meta-analyses indicating that appropriate antimicrobial therapy has survival benefit in both BSI [22] and severe sepsis [23, 24]. Our data shows that delays in administration of appropriate antimicrobials impact on outcome in BSI over days 0–6.

### Strengths and limitations

This is one of the largest observational cohort studies of patients with BSI in the NHS. A key strength of the study is that it did not require individual patient consent therefore reducing the risk of selection bias. For example, less acutely ill patients may be more likely to be approached and consent which could undermine the scientific integrity and public value of the research. The National Information Governance Board approved the use of such routinely collected data without individual patient consent under section 251 of the NHS Act 2006. A large number of data items were collected which enabled us adequality control for potential confounding in the analysis and also allowed us to include variables that are predictive of missing data in a covariate of interest in the multiple imputation procedure making the missing at random assumption plausible. There are some limitations of the study. Firstly, focussing on six key pathogens may limit the generalisability of the results. This study focused on patients with clinically significant pathogens that produce large numbers of infections and may have multidrug resistance. We specifically focused on pathogens that were highly unlikely to be contaminants but were also common cases of BSI, linked with significant mortality and remain a significant problem across the NHS. Results may not be generalisable to other bloodstream infections and therefore conclusions drawn should be limited to the six key pathogens studied. Finally, this study focussed on NHS-employed nurses, health- care assistants and agency nurses, but did not explore the impact of other medical staffing levels such as consultants which may merit analysis in future research.

## Conclusions

Our overall conclusion is that for all the pathogen groups studied timely appropriate antimicrobial therapy was associated with improved clinical outcome as measured by mortality over 28 days, and the effect of each day delay was most marked in the first 7 days. In addition, increased ward activity (admissions and discharges) was associated with increased hazard of death within medical wards and especially in critical care wards.

## Supplementary information

**Additional file 1: Supplemental Table 1.** Non-modifiable risk factors ^a^. **Supplemental Table 2.** Modifiable risk factors ^a^. **Supplemental Table 3.** Summary table of additional non-modifiable risk factors, by 28-day survival status. **Supplemental Table 4.** Lines and urinary catheter details at day/time 0, by 28-day survival status. **Supplemental Table 5.** Appropriate antimicrobial therapy, by organism and survival. **Supplemental Table 6.** Multivariable Cox model of non-modifiable risk factors on 28-day mortality ^a^. **Supplemental Table 7.** Univariable and multivariable Cox model of modifiable risk factors on 28-day mortality. **Supplemental Table 8.** Predicted and observed risk by decile of predicted risk. **Supplemental Table 9.** Mortality at 7-days and 28 days, by organism. **Supplemental Table 10.** Univariable and multivariable Cox model of modifiable risk factors on 7-day mortality. **Supplemental Figure 1.** Flow of participant samples. **Supplemental Figure 2.** Average number of staff per 10 beds, by day of week. **Supplemental Figure 3.** Average ward activity per 10 beds, by day of week. **Supplemental Figure 4.** Sensitivity analysis of primary outcome: removal of “36 hour rule” in definition of time to appropriate therapy. **Supplemental Figure 5.** Sensitivity analysis of primary outcome: 12-h (in place of 36-h) rule in definition of time to appropriate therapy. **Supplemental Figure 6.** Sensitivity analysis of primary outcome: 24-h (in place of 36-h) rule in definition of time to appropriate therapy. **Supplemental Figure 7.** Sensitivity analysis of primary outcome: Complete case analysis.

## Data Availability

The datasets used and/or analysed during the current study are available from the corresponding author on reasonable request.

## Abbreviations

BP: Blood pressure
BSI: Bloodstream infection
BSI-FOO: Bloodstream Infections – Focus on Outcomes
CDC: Centres for Disease Control and Prevention
CI: Confidence interval
CKD: Chronic kidney disease
COPD: Chronic obstructive pulmonary disease
eGFR: Estimated glomerular filtration rate
ESBL: Extended-spectrum beta-lactamase
HCA: Healthcare assistant
HR: Hazard ratio
ICU: Intensive care unit
IQR: Interquartile range
MRSA: Methicillin-resistant *S. aureus*
MSSA: Methicillin-susceptible *S. aureus*
NHS: National Health Service
OR: Odds Ratio
SD: Standard deviation
