# Supplementary material for: Modifiable healthcare factors affecting 28-day survival in bloodstream infection: a prospective cohort study
Source: BMC Infect Dis. 2020 Jul 25;20:545. doi: 10.1186/s12879-020-05262-6 (PMC7382856; doi:10.1186/s12879-020-05262-6)
Supplement: Supplementary file 1 — Additional file 1: Supplemental Table 1. Non-modifiable risk factors a. Supplemental Table 2. Modifiable risk factors a. Supplemental Table 3. Summary table of additional non-modifiable risk factors, by 28-day survival status. Supplemental Table 4. Lines and urinary catheter details at day/time 0, by 28-day survival status. Supplemental Table 5. Appropriate antimicrobial therapy, by organism and survival. Supplemental Table 6. Multivariable Cox model of non-modifiable risk factors on 28-day mortality a. Supplemental Table 7. Univariable and multivariable Cox model of modifiable risk factors on 28-day mortality. Supplemental Table 8. Predicted and observed risk by decile of predicted risk. Supplemental Table 9. Mortality at 7-days and 28 days, by organism. Supplemental Table 10. Univariable and multivariable Cox model of modifiable risk factors on 7-day mortality. Supplemental Figure 1. Flow of participant samples. Supplemental Figure 2. Average number of staff per 10 beds, by day of week. Supplemental Figure 3. Average ward activity per 10 beds, by day of week. Supplemental Figure 4. Sensitivity analysis of primary outcome: removal of “36 hour rule” in definition of time to appropriate therapy. Supplemental Figure 5. Sensitivity analysis of primary outcome: 12-h (in place of 36-h) rule in definition of time to appropriate therapy. Supplemental Figure 6. Sensitivity analysis of primary outcome: 24-h (in place of 36-h) rule in definition of time to appropriate therapy. Supplemental Figure 7. Sensitivity analysis of primary outcome: Complete case analysis. [file 12879_2020_5262_MOESM1_ESM.docx]

**Modifiable healthcare factors affecting outcome in bloodstream infection: the BSI-FOO prospective cohort study**

**Additional File 1**

**Supplemental tables**

[**Supplemental table 1** Non-modifiable risk factors ^a^ 2](#_Toc44319515)

[**Supplemental table 2** Modifiable risk factors ^a^ 4](#_Toc44319516)

[**Supplemental table 3** Summary table of additional non-modifiable risk factors, by 28-day survival status 6](#_Toc44319517)

[**Supplemental table 4** Lines and urinary catheter details at day/time 0, by 28-day survival status 8](#_Toc44319518)

[**Supplemental table 5** Appropriate antimicrobial therapy, by organism and survival 9](#_Toc44319519)

[**Supplemental table 6** Multivariable Cox model of non-modifiable risk factors on 28-day mortality ^a^ 12](#_Toc44319520)

[**Supplemental table 7** Univariable and multivariable Cox model of modifiable risk factors on 28-day mortality………………………………. 14](#_Toc44319521)

[**Supplemental table 8** Predicted and observed risk by decile of predicted risk 16](#_Toc44319522)

[**Supplemental table 9** Mortality at 7-days and 28 days, by organism 17](#_Toc44319523)

[**Supplemental table 10** Univariable and multivariable Cox model of modifiable risk factors on 7-day mortality………………………………... 18](#_Toc44319524)

**Supplemental figures**

[**Supplemental figure 1** Flow of participant samples 5](#_Toc44319652)

[**Supplemental figure 2** Average number of staff per 10 beds, by day of week 10](#_Toc44319653)

[**Supplemental figure 3** Average ward activity per 10 beds, by day of week 11](#_Toc44319654)

[**Supplemental figure 4** Sensitivity analysis of primary outcome: removal of "36 hour rule" in definition of time to appropriate therapy………… 20](#_Toc44319655)

[**Supplemental figure 5** Sensitivity analysis of primary outcome: 12-hour (in place of 36-hour) rule in definition of time to appropriate therapy 21](#_Toc44319656)

[**Supplemental figure 6** Sensitivity analysis of primary outcome: 24-hour (in place of 36-hour) rule in definition of time to appropriate therapy 22](#_Toc44319657)

[**Supplemental figure 7** Sensitivity analysis of primary outcome: Complete case analysis 23](#_Toc44319658)

**Supplemental table 1** Non-modifiable risk factors ^a^

| **Type** | **Factors** |
| --- | --- |
| Organisational | Centre  Admission from nursing or care home  Length of prior in-patient stay (days)  Speciality of consultant on day 0 ^b^ |
| Organism / infection | Organism identity (target organism group)  Source of infection (CDC criteria) |
| Patient measures | Age  Gender  Height (cm)  Weight (kg) |
| Patient medical history  (up to date 0) | Leukaemia within 5 years before date 0  Lymphoma within 5 years before date 0  Solid tumour within 5 years before date 0  Any other (second) tumour within 5 years before date 0  Chemotherapy in month before date 0  Surgery requiring overnight stay within 7 days before date 0  Burn requiring hospital admission within 7 days before date 0  Cardiac arrest within 7 days before date 0  Myocardial infarction, symptomatic within 7 days before date 0  Renal support within 7 days before date 0 |
| Patient comorbidities ongoing at date 0 | Disease markers  Ascites  Diabetes without organ damage  Diabetes with organ damage  Chronic obstructive pulmonary disease  Congestive heart failure  Connective tissue disease  Cerebrovascular disease  Dementia  Hemiplegia  Peptic ulcer disease  Peripheral vascular disease  Potentially removable sources of infection  Abscess at time 0  Infected foreign body (non-surgical) at time 0  Infected prosthesis or similar surgical item at time 0 |
| Infection severity measures at or nearest before time 0 | Signs  Mental Disorientation (scale 0-4) at time 0  Temperature (°C) at time 0  Systolic blood pressure (mmHg) at time 0  Early warning score at time 0  Blood tests  INR^c^ at day0, or nearest within 7 days before  eGFR^d^ (mL/min/1.73 m^2^) at day 0, or nearest within 7 days before  Serum albumin (g/L) at day0, or nearest within 7 days before  Bilirubin (total, micromol/L) at day0, or nearest within 7 days before  Neutrophil count (×10^9^/L) at day 0, or nearest within 7 days before  Interventions  Receiving intravenous fluids on day 0, at or before time 0  Receiving artificial ventilation on day 0, at or before time 0  Receiving vasopressor drugs on day 0, at or before time 0  Received systemic corticosteroids in 24 hours before time 0 |

^a^ All variables listed in this table were considered for potential inclusion in the adjusted analysis of non-modifiable risk factors used to derive the risk score, unless otherwise stated.

^b^ Speciality of consultant on day 0 was not included as a potential covariate in modelling as it was correlated with day 0 ward speciality, which was of more interest.

^c^ INR: international normalised ratio (a measure of blood clotting speed related to liver function)

^d^ eGFR: estimated glomerular filtration rate (a measure of kidney function).

**Supplemental table 2** Modifiable risk factors ^a^

| **Risk factor** | **Definition** | **Detail** |
| --- | --- | --- |
| Ward speciality ^b^ | Medicine, Major surgery, Minor surgery, Critical care or Other | Observed each day, days 0–7 |
| Staffing per 10 beds (nursing and care staff) | Average number of staff (NHS-employed nurses + agency nurses + healthcare assistants) over the 3 shifts, per 10 beds | Observed each day, days 0–7 |
| Ward activity per 10 beds | Number of patients admitted to ward + number of patients discharged from ward, per 10 beds | Observed each day, days 0–7 |
| Central vascular line | Central line present, yes or no | Determined ^c^ each day, days 0–28 |
| Peripheral vascular line | Peripheral line present, yes or no | Determined ^c^ each day, days 0–28 |
| Urinary catheter | Urinary catheter present, yes or no | Determined ^c^ each day, days 0–28 |
| Ward movement: to critical care | Cumulative count of moves from a critical care ward to a medical or surgical ward | Total number of relevant ward moves up to and including that day, for days 0–7 |
| Ward movement: from critical care | Cumulative count of moves from a critical care ward to a medical or surgical ward | Total number of relevant ward moves up until that day, for days 0–7 |
| Ward movement: within speciality | Cumulative count of ward moves within the same speciality (surgery, medicine or critical care) | Total number of relevant ward movements up until that day, for days 0–7 |
| Ward movement: from medicine to surgery | Cumulative count of moves from a medical to a surgical ward | Total number of relevant ward movements up until that day, for days 0–7 |
| Ward movement: from surgery to medicine | Cumulative count of moves from a surgical to a medical ward | Total number of relevant ward movements up until that day, for days 0–7 |
| Time to initiation of appropriate antimicrobial therapy | Cumulative count of days before first receipt of appropriate antimicrobial therapy | Total number of days before first appropriate therapy up until that day, for days 0–28 |
| Duration of first appropriate antimicrobial therapy ^d^ | Cumulative count of days on which first appropriate therapy was received | Total number of days when first appropriate therapy was received up until that day, for days 0–28. (Zero before day of first dose of appropriate therapy.) |

^a^ All variables listed in this table were considered for potential inclusion in the adjusted analysis of modifiable risk factors, unless otherwise stated.

^b^ For statistical modelling purposes, ward specialities were grouped as medicine, surgery (minor surgery + major surgery) and critical care; ward specialities in the “Other” category were included in either surgery (obstetrics & gynaecology) or medicine (A&E, emergency assessment, fracture clinics and related units, imaging, diagnostics and telemetry, and other services not already classified as medical, surgical or HDU/ITU).

^c^ Determined from presence/absence of line/catheter on day0 and date of removal.

^d^ Duration of appropriate antimicrobial therapy was excluded as it was bounded by survival time and highly correlated with time to receipt of appropriate antimicrobial therapy, therefore including both led to biased and/or uninterpretable result

**Supplemental figure 1** Flow of participant samples
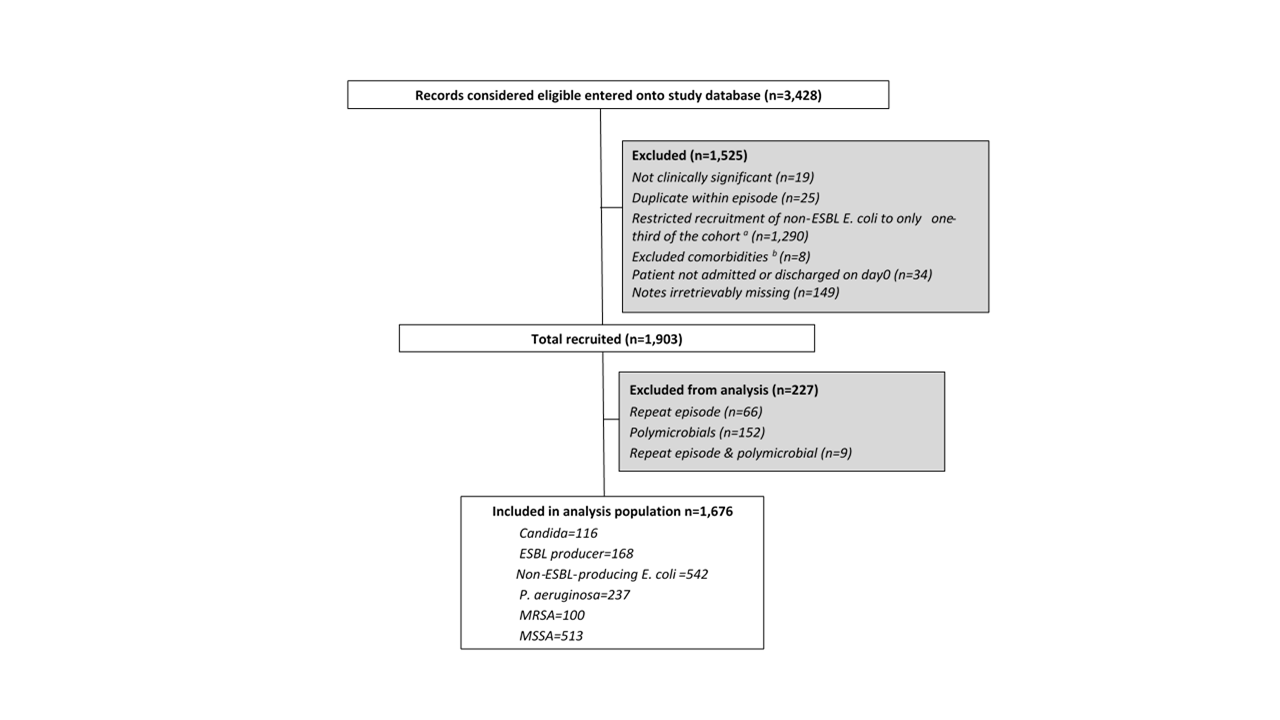


*^a^ Episodes of non-ESBL-producing E. coli were presented for randomisation after being entered to the study database and randomisation was stratified by centre and blocked using random block sizes.*

*^b^ Excluded comorbidities: Having Cystic Fibrosis, Human Immunodeficiency Virus (HIV) positive, Patients on the end of life pathway*

***Abbreviations:*** *ESBL= Extended-spectrum beta-lactamase, MRSA- Methicillin-resistant S. aureus, MSSA= Methicillin-susceptible S. aureus*

**Supplemental table 3**  Summary table of additional non-modifiable risk factors, by 28-day survival status

| **Risk factor** | **Survived (n=1,328)** | | **Died (n=348)** | | **Overall (n=1,676)** | |
| --- | --- | --- | --- | --- | --- | --- |
|  | **n** | **%** | **n** | **%** | **n** | **%** |
| **Patient medical history** |  |  |  |  |  |  |
| Leukaemia within last 5 years | 90/1328 | 6.8% | 34/348 | 9.8% | 124/1676 | 7.4% |
| Lymphoma within last 5 years | 75/1328 | 5.6% | 20/348 | 5.7% | 95/1676 | 5.7% |
| Solid tumour within last 5 years | 258/1328 | 19.4% | 101/348 | 29.0% | 359/1676 | 21.4% |
| Any other tumour within last 5 years | 60/1327 | 4.5% | 22/348 | 6.3% | 82/1675 | 4.9% |
| Surgery (overnight stay) ≤7 days before date 0 | 118/1327 | 8.9% | 34/348 | 9.8% | 152/1675 | 9.1% |
| If yes: |  |  |  |  |  |  |
| Elective surgery | 77/118 | 65.3% | 14/34 | 41.2% | 91/152 | 59.9% |
| Surgical speciality |  |  |  |  |  |  |
| Cardiothoracic surgery | 13/118 | 11.0% | 4/34 | 11.8% | 17/152 | 11.2% |
| General surgery | 59/118 | 50.0% | 19/34 | 55.9% | 78/152 | 51.3% |
| Neurosurgery | 14/118 | 11.9% | 6/34 | 17.6% | 20/152 | 13.2% |
| Plastic surgery | 2/118 | 1.7% | 2/34 | 5.9% | 4/152 | 2.6% |
| Trauma & orthopaedics | 8/118 | 6.8% | 0/34 | 0.0% | 8/152 | 5.3% |
| Urology | 16/118 | 13.6% | 3/34 | 8.8% | 19/152 | 12.5% |
| Ear nose & throat | 2/118 | 1.7% | 0/34 | 0.0% | 2/152 | 1.3% |
| Oral & maxillo facial surgery | 1/118 | 0.8% | 0/34 | 0.0% | 1/152 | 0.7% |
| Obstetrics and gynaecology | 3/118 | 2.5% | 0/34 | 0.0% | 3/152 | 2.0% |
| Body area of surgery |  |  |  |  |  |  |
| Superficial | 4/117 | 3.4% | 3/34 | 8.8% | 7/151 | 4.6% |
| Head & neck | 17/117 | 14.5% | 6/34 | 17.6% | 23/151 | 15.2% |
| Upper limbs | 4/117 | 3.4% | 0/34 | 0.0% | 4/151 | 2.6% |
| Lower limbs | 7/117 | 6.0% | 2/34 | 5.9% | 9/151 | 6.0% |
| Thoracic cavity | 16/117 | 13.7% | 4/34 | 11.8% | 20/151 | 13.2% |
| Abdominal cavity | 69/117 | 59.0% | 19/34 | 55.9% | 88/151 | 58.3% |
| Abscess at time 0 | 96/1327 | 7.2% | 12/347 | 3.5% | 108/1674 | 6.5% |
| Surgical prosthesis time 0 | 19/1327 | 1.4% | 3/347 | 0.9% | 22/1674 | 1.3% |

**Abbreviations:** SD=Standard deviation, IQR =Interquartile range

**Supplemental table 4**  Lines and urinary catheter details at day/time 0, by 28-day survival status

| **Line detail** |  | **Survived (n=1,328)** | | **Died (n=348)** | | **Overall (n=1,676)** | |
| --- | --- | --- | --- | --- | --- | --- | --- |
|  |  | **n** | **%** | **n** | **%** | **n** | **%** |
| **Lines:** |  |  |  |  |  |  |  |
| Any line (central or peripheral) present at time 0 |  | 821/1326 | 61.9% | 262/347 | 75.5% | 1083/1673 | 64.7% |
| Central line present at time 0 |  | 300/1326 | 22.6% | 106/347 | 30.5% | 406/1673 | 24.3% |
| Number of days from day 0 to central line removal ^a^ | Median (IQR) | 3.0 | (1.0, 6.0) | 2.0 | (1.0, 4.0) | 2.0 | (1.0, 6.0) |
| Central line implicated infection ^b^ |  | 123/1327 | 9.3% | 15/348 | 4.3% | 138/1675 | 8.2% |
| Number of days from day 0 to central line removal for those with a central line implicated infection ^c^ |  | 2.0 | (1.0, 4.0) | 2.0 | (1.0, 3.0) | 2.0 | (1.0, 4.0) |
| Peripheral line present at time 0 |  | 621/1326 | 46.8% | 217/347 | 62.5% | 838/1673 | 50.1% |
| Number of days to peripheral line removal from day 0 ^d^ | Median (IQR) | 1.0 | (1.0, 3.0) | 1.0 | (1.0, 2.0) | 1.0 | (1.0, 3.0) |
| Peripheral line implicated infection ^b^ |  | 20/1327 | 1.5% | 7/348 | 2.0% | 27/1675 | 1.6% |
| Number of days to peripheral line removal for those with a peripheral line implicated infection ^e^ |  | 0.0 | (0.0, 2.0) | 1.5 | (0.0, 2.0) | 0.0 | (0.0, 2.0) |
| **Urinary catheters:** |  |  |  |  |  |  |  |
| Urinary catheter present at time 0 |  | 357/1326 | 26.9% | 167/347 | 48.1% | 524/1673 | 31.3% |
| Number of days to urinary catheter removal from day 0 ^f^ | Median (IQR) | 4.0 | (1.0, 10.0) | 4.0 | (1.0, 8.0) | 4.0 | (1.0, 8.0) |

^a^ Data missing for 82 patients (59 survived, 23 died)

^b^ Source of infection was recorded using CDC criteria

^c^ Data missing for 13 patients (13 survived, 0 died)

^d^ Data missing for 190 patients (141 survived, 149 died)

^e^ Data missing for 2 patients (1 survived, 1 died)

^f^ Data missing for 178 patients (132 survived, 46 died)

***Abbreviations:*** *IQR =Interquartile range, CDC= Centres for Disease Control and Prevention*

**Supplemental table 5** Appropriate antimicrobial therapy, by organism and survival

| **Organism** |  | **Survived (n=1,328)** | | **Died (n=348)** | | **Overall (n=1,676)** | |
| --- | --- | --- | --- | --- | --- | --- | --- |
|  |  | **n** | **%** | **n** | **%** | **n** | **%** |
| Non-ESBL *E. coli* | Received appropriate therapy * | 422/470 | 89.8% | 52/72 | 72.2% | 474/542 | 87.5% |
|  | If Yes, median (IQR) time to receipt (hours) | 5.5 | (1.0, 27.0) | 3.5 | (0.0, 16.5) | 5.0 | (1.0, 26.0) |
|  | If Yes, median (IQR) duration of therapy (hours) | 157.0 | (107.0, 212.0) | 164.5 | (98.0, 223.5) | 158.0 | (106.0, 212.0) |
| ESBL producer | Received appropriate therapy * | 123/134 | 91.8% | 19/34 | 55.9% | 142/168 | 84.5% |
|  | If Yes, median (IQR) time to receipt (hours) | 15.0 | (1.0, 48.0) | 28.0 | (1.0, 51.0) | 19.0 | (1.0, 48.0) |
|  | If Yes, median (IQR) duration of therapy (hours) | 169.0 | (137.0, 222.0) | 134.0 | (73.0, 184.0) | 167.0 | (132.0, 218.0) |
| *Candida* | Received appropriate therapy * | 60/82 | 73.2% | 11/34 | 32.4% | 71/116 | 61.2% |
|  | If Yes, median (IQR) time to receipt (hours) | 75.5 | (45.5, 121.5) | 60.0 | (48.0, 96.0) | 74.0 | (46.0, 115.0) |
|  | If Yes, median (IQR) duration of therapy (hours) | 301.5 | (120.0, 366.0) | 158.0 | (69.0, 234.0) | 240.0 | (119.0, 349.0) |
| MRSA | Received appropriate therapy * | 67/71 | 94.4% | 13/29 | 44.8% | 80/100 | 80.0% |
|  | If Yes, median (IQR) time to receipt (hours) | 37.0 | (7.0, 69.0) | 29.0 | (3.0, 50.0) | 35.5 | (5.5, 66.0) |
|  | If Yes, median (IQR) duration of therapy (hours) | 279.0 | (138.0, 590.0) | 126.0 | (74.0, 170.0) | 197.5 | (117.5, 420.5) |
| MSSA | Received appropriate therapy * | 386/406 | 95.1% | 72/107 | 67.3% | 458/513 | 89.3% |
|  | If Yes, median (IQR) time to receipt (hours) | 5.0 | (1.0, 29.0) | 5.5 | (1.0, 31.5) | 5.0 | (1.0, 30.0) |
|  | If Yes, median (IQR) duration of therapy (hours) | 312.0 | (184.0, 496.0) | 169.5 | (90.5, 273.5) | 287.0 | (165.0, 440.0) |
| *P. aeruginosa* | Received appropriate therapy * | 151/165 | 91.5% | 40/72 | 55.6% | 191/237 | 80.6% |
|  | If Yes, median (IQR) time to receipt (hours) | 4.0 | (0.0, 35.0) | 9.5 | (1.5, 32.0) | 5.0 | (1.0, 33.0) |
|  | If Yes, median (IQR) duration of therapy (hours) | 162.0 | (121.0, 230.0) | 116.5 | (79.5, 178.0) | 158.0 | (113.5, 220.0) |
| **Overall** | **Received appropriate therapy *** | **1209/1328** | **91.0%** | **207/348** | **59.5%** | **1416/1676** | **84.5%** |
|  | **If Yes, median (IQR) time to receipt (hours)** | **7.0** | **(1.0, 40.0)** | **8.0** | **(1.0, 38.0)** | **7.0** | **(1.0, 40.0)** |
|  | **If Yes, median (IQR) duration of therapy (hours)** | **192.0** | **(126.0, 319.0)** | **146.0** | **(88.0, 224.0)** | **183.0** | **(119.5, 304.5)** |

^*^ Therapy was defined as appropriate if susceptible and received for at least 36 hours

**Abbreviations:** ESBL= Extended-spectrum beta-lactamase, MRSA- Methicillin-resistant S. aureus, MSSA= Methicillin-susceptible S. aureus, IQR=Interquartile range

**Supplemental figure 2** Average number of staff per 10 beds, by day of week


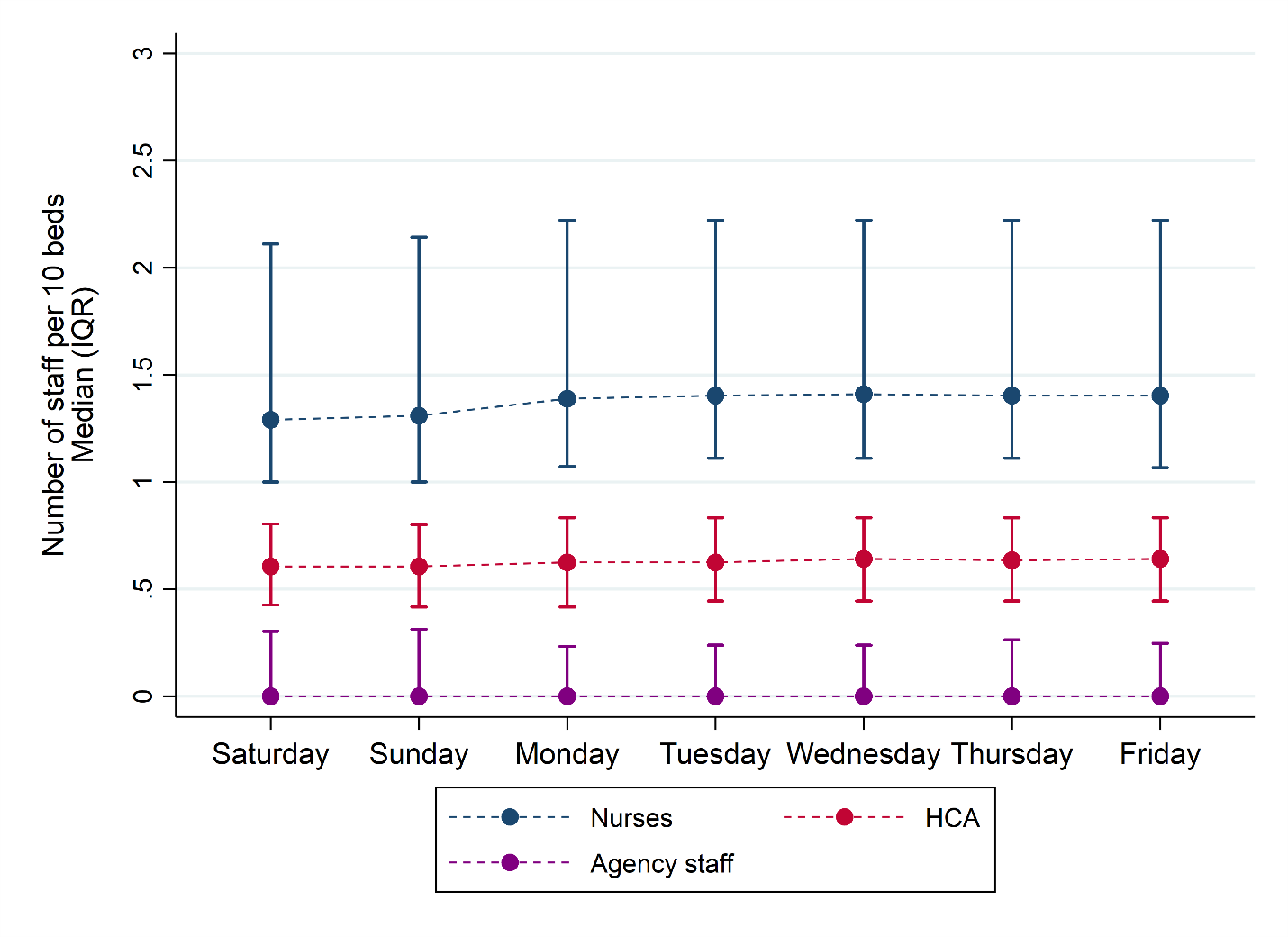


***Abbreviations:*** *HCA=Healthcare assistant, IQR=Interquartile range*

**Supplemental figure 3** Average ward activity per 10 beds, by day of week


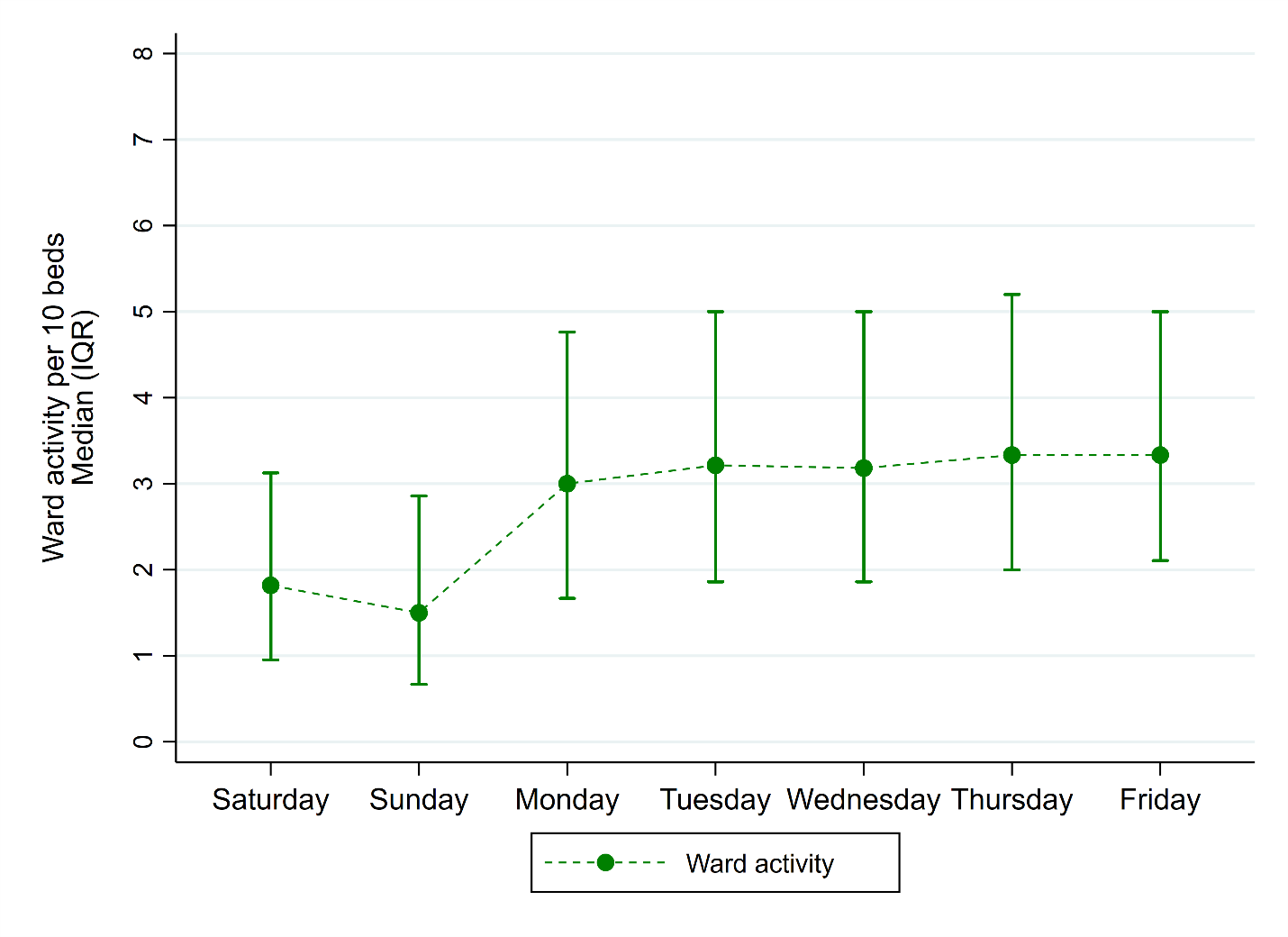


**Abbreviations:** IQR=Interquartile range

**Supplemental table 6** Multivariable Cox model of non-modifiable risk factors on 28-day mortality ^a^

| **Risk factor** |  | **Hazard ratio** | **95% CI** | **P value** |
| --- | --- | --- | --- | --- |
| Age (years) |  | 1.02 | (1.01, 1.03) | <0.001 |
| Temperature at time 0 (°C) |  | 0.83 | (0.75, 0.92) | <0.001 |
| Weight (kg) |  | 0.99 | (0.98, 1.00) | 0.136 |
| Systolic BP at day 0 or closest (mmHg) |  | 1.43 | (1.17, 1.75) | <0.001 |
| Admission from nursing home |  | 1.36 | (0.94, 1.98) | 0.104 |
| Serum albumin (g/L) |  | 0.94 | (0.92, 0.96) | <0.001 |
| Bilirubin total (µmol/L) |  | 1.00 | (1.00, 1.00) | 0.188 |
| Renal support within 7 days before date 0 |  | 1.53 | (1.01, 2.31) | 0.044 |
| On ventilation at day 0 |  | 1.33 | (0.94, 1.90) | 0.111 |
| On intravenous fluids at day 0 |  | 1.21 | (0.96, 1.53) | 0.105 |
| Systemic corticosteroids in last 24 hours |  | 1.75 | (1.35, 2.28) | <0.001 |
| Abscess at time 0 |  | 0.63 | (0.34, 1.15) | 0.133 |
| Congestive heart failure |  | 1.35 | (1.00, 1.84) | 0.053 |
| Peripheral vascular disease |  | 1.50 | (1.06, 2.12) | 0.021 |
| Cerebrovascular disease |  | 1.32 | (1.00, 1.74) | 0.050 |
| Peptic ulcer disease |  | 1.38 | (0.93, 2.05) | 0.108 |
| Ascites |  | 2.04 | (1.33 ,3.12) | 0.001 |
| Leukaemia within last 5 years |  | 2.01 | (1.36, 2.96) | <0.001 |
| Solid tumour within last 5 years |  | 1.38 | (1.07, 1.79) | 0.015 |
| Any other tumour within last 5 years |  | 1.54 | (0.97, 2.45) | 0.070 |
| Mental disorientation: | *None* | 1 | - | 0.136 |
|  | Grade I | 0.89 | (0.35, 1.44) |  |
|  | Grade II | 1.54 | (1.08, 2.19) |  |
|  | Grade III | 1.01 | (0.61, 1.67) |  |
|  | Grade IV | 1.40 | (0.64, 3.06) |  |
| eGFR | *Normal/Stage 1* | 1 | - | 0.007 |
|  | Stage 2 CKD^1^ | 0.94 | (0.66, 1.33) |  |
|  | Stage 3 CKD | 0.88 | (0.61, 1.27) |  |
|  | Stage 4 CKD | 1.55 | (1.06, 2.26) |  |
|  | Stage 5 CKD | 0.82 | (0.48, 1.42) |  |
| Source of infection | *Gastrointestinal system* | 1 | - | <0.001 |
|  | Line | 1.62 | (0.83, 3.15) |  |
|  | Lower respiratory tract | 4.71 | (2.58, 8.58) |  |
|  | Skin and surgical site | 1.73 | (0.89, 3.37) |  |
|  | Systemic & site uncertain | 3.37 | (1.96, 5.78) |  |
|  | Urinary tract | 1.29 | (0.73, 2.30) |  |
|  | Other | 1.56 | (0.74, 3.30) |  |

^a^ Model adjusted for centre

**Abbreviations:** BP=Blood pressure, eGFR=Estimated glomerular filtration rate, CKD=Chronic kidney disease

**Supplemental table 7** Univariable and multivariable Cox model of modifiable risk factors on 28-day mortality

| **Variable** |  |  | | **Univariable** | | **Multivariable** | |
| --- | --- | --- | --- | --- | --- | --- | --- |
|  |  |  | | **Hazard ratio (95% CI)** | **P-value** | **Hazard ratio (95% CI)** | **P-value** |
| Risk score |  | Within medicine | | 3.11 (2.64 – 3.66) | <0.001 | 2.77 (2.35 – 3.27) | <0.001 |
|  |  | Within critical care | | 1.80 (1.53 – 2.11) | <0.001 | 1.84 (1.54 – 2.19) | <0.001 |
|  |  | Within surgery | | 2.81 (2.12 – 3.73) | <0.001 | 2.89 (2.13 – 3.90) | <0.001 |
| Organism ^a^ | *Non-ESBL E. coli* |  |  | *Reference* | <0.001 | *Reference* | 0.369 |
|  | ESBL producer |  |  | 1.61 (1.07 – 2.43) |  | 1.10 (0.70 – 1.73) |  |
|  | *Candida* |  |  | 2.47 (1.64 - 3.72) |  | 0.57 (0.32 – 1.02) |  |
|  | MRSA |  |  | 2.44 (1.59 – 3.76) |  | 1.23 (0.74 – 2.04) |  |
|  | MSSA |  |  | 1.65 (1.22 – 2.22) |  | 1.40 (1.00 – 1.95) |  |
|  | *P. aeruginosa* |  |  | 2.59 (1.87 – 3.59) |  | 1.20 (0.83 – 1.74) |  |
| Ward speciality ^b^ | *Medicine* |  |  | *Reference* | <0.001 | *Reference* | <0.001 |
|  | Critical care |  |  | 2.52 (1.98 – 3.21) |  | 2.11 (1.30 – 3.41) |  |
|  | Surgery |  |  | 0.52 (0.37 – 0.73) |  | 0.63 (0.39 – 1.02) |  |
| Central line present |  |  | | 1.86 (1.34 – 2.58) | <0.001 | 1.08 (0.75 – 1.56) | 0.674 |
| Peripheral line present |  |  | | 1.65 (1.14 – 2.38) | 0.008 | 1.12 (0.77 – 1.63) | 0.562 |
| Urinary catheter present |  |  |  | 2.66 (2.04 – 3.47) | <0.001 | 1.14 (0.84 – 1.56) | 0.394 |
| Average number of staff across the 3 shifts, per 10 beds |  | Within medicine | | 1.09 (0.96 -1.23) | 0.176 | 1.00 (0.86 – 1.16) | 0.982 |
|  |  | Within critical care | | 1.02 (0.97 – 1.06) | 0.458 | 0.99 (0.96 – 1.02) | 0.580 |
|  |  | Within surgery | | 1.12 (0.75 – 1.66) | 0.579 | 0.95 (0.63 – 1.44) | 0.810 |
| Ward activity (number of admissions and discharges) per 10 beds |  | Within medicine | | 1.03 (1.01 – 1.06) | 0.001 | 1.04 (1.01 – 1.06) | 0.001 |
|  |  | Within critical care | | 1.07 (1.02 – 1.13) | 0.008 | 1.12 (1.06 – 1.19) | <0.001 |
|  |  | Within surgery | | 0.96 (0.85 – 1.08) | 0.478 | 1.03 (0.91 – 1.16) | 0.643 |
| Movement within ward speciality | One or more vs none |  |  | 0.61 (0.45 – 0.83) | 0.002 | 0.67 (0.48 – 0.93) | 0.016 |
| Movement to critical care | One or more vs none |  |  | 1.64 (1.12 – 2.39) | 0.010 | 1.32 (0.84 – 2.07) | 0.228 |
| Movement from critical care | One or more vs none |  |  | 0.47 (0.23 – 0.95) | 0.035 | 0.52 (0.24 – 1.11) | 0.090 |
| Movement from surgery to medicine | One or more vs none |  |  | 0.91 (0.38 – 2.21) | 0.840 | 1.22 (0.45 – 3.33) | 0.692 |
| Movement from medicine to surgery | One or more vs none |  |  | 0.42 (0.20 – 0.89) | 0.024 | 0.93 (0.39 – 2.20) | 0.868 |
| Time to appropriate therapy (days) |  | days 0–6 | Within non-ESBL *E. coli* | 1.62 (1.32 – 2.00) | <0.001 | 1.45 (1.15 – 1.83) | 0.001 |
|  |  |  | Within ESBL producer | 1.80 (1.39 – 2.33) | <0.001 | 1.84 (1.43 – 2.37) | <0.001 |
|  |  |  | Within *Candida* | 1.65 (1.30 – 2.09) | <0.001 | 1.62 (1.27 – 2.05) | <0.001 |
|  |  |  | Within MRSA | 1.57 (1.17 – 2.10) | 0.002 | 1.39 (1.03 – 1.88) | 0.033 |
|  |  |  | Within MSSA | 1.96 (1.68 – 2.30) | <0.001 | 2.02 (1.71 – 2.38) | <0.001 |
|  |  |  | Within *P. aeruginosa* | 1.92 (1.63 – 2.27) | <0.001 | 1.69 (1.42 – 2.01) | <0.001 |
|  |  | days 7–13 | Within non-ESBL *E. coli* | 0.89 (0.72 – 1.10) | 0.276 | 0.87 (0.68 – 1.11) | 0.268 |
|  |  |  | Within ESBL producer | 1.09 (0.95 – 1.26) | 0.218 | 1.13 (0.98 – 1.31) | 0.102 |
|  |  |  | Within *Candida* | 0.95 (0.80 – 1.12) | 0.525 | 0.94 (0.79 – 1.12) | 0.522 |
|  |  |  | Within MRSA | 1.10 (0.97 - 1.25) | 0.127 | 1.05 (0.92 – 1.21) | 0.475 |
|  |  |  | Within MSSA | 0.93 (0.77 – 1.12) | 0.423 | 0.97 (0.78 – 1.20) | 0.754 |
|  |  |  | Within *P. aeruginosa* | 0.79 (0.55 – 1.13) | 0.193 | 0.74 (0.48 – 1.13) | 0.166 |
|  |  | days 14–28 | Within non-ESBL *E. coli* | 0.69 (0.35 – 1.34) | 0.271 | 0.60 (0.29 – 1.23) | 0.160 |
|  |  |  | Within ESBL producer | 0.92 (0.72 – 1.17) | 0.503 | 0.93 (0.71 – 1.22) | 0.587 |
|  |  |  | Within *Candida* | 1.03 (0.97 – 1.09) | 0.363 | 1.03 (0.97 – 1.11) | 0.318 |
|  |  |  | Within MRSA | 0.99 (0.87 – 1.13) | 0.874 | 0.95 (0.82 – 1.11) | 0.541 |
|  |  |  | Within MSSA | 1.01 (0.95 – 1.08) | 0.709 | 1.06 (0.99 – 1.14) | 0.100 |
|  |  |  | Within *P. aeruginosa* | 1.01 (0.95 – 1.08) | 0.673 | 1.02 (0.95 – 1.09) | 0.655 |

**Notes:**

^a^ Effect of organism is given for the time period 0 to 6 days, when time to appropriate therapy is 1 day (multivariable model)

^b^ Effect of ward speciality is given for the median number of staff per 10 beds, median ward activity and median risk score (multivariable model)

**Supplemental table 8** Predicted and observed risk by decile of predicted risk

| **Decile of predicted risk** | **Observed risk** | **Predicted risk*** |
| --- | --- | --- |
| 1 | 0.01 | 0.01 |
| 2 | 0.07 | 0.02 |
| 3 | 0.08 | 0.03 |
| 4 | 0.09 | 0.05 |
| 5 | 0.15 | 0.07 |
| 6 | 0.18 | 0.11 |
| 7 | 0.21 | 0.15 |
| 8 | 0.28 | 0.22 |
| 9 | 0.32 | 0.34 |
| 10 | 0.29 | 0.65 |

* Mean predicted risk within each risk decile

**Supplemental table 9** Mortality at 7-days and 28 days, by organism

| **Organism** | **Died at 7 days** | | **Died at 28 days** | |
| --- | --- | --- | --- | --- |
|  | **n** | **%** | **n** | **%** |
| MRSA (n=100) | 14 | 14.0% | 29 | 29.0% |
| MSSA (n=513) | 54 | 10.5% | 107 | 20.9% |
| Non-ESBL *E. coli* (n=542) | 28 | 5.2% | 72 | 13.3% |
| ESBL producer (n=168) | 19 | 11.3% | 34 | 20.2% |
| *Candida* (n=116) | 21 | 18.1% | 34 | 29.3% |
| *P. aeruginosa* (n=237) | 49 | 20.7% | 72 | 30.4% |
| **Overall (n=1676)** | **185** | **11.0%** | **348** | **20.8%** |

***Abbreviations:*** *ESBL= Extended-spectrum beta-lactamase, MRSA- Methicillin-resistant S. aureus, MSSA= Methicillin-susceptible S. aureus*

**Supplemental table 10** Univariable and multivariable Cox model of modifiable risk factors on 7-day mortality

| **Variable** |  |  | | **Univariable** | | **Multivariable** | |
| --- | --- | --- | --- | --- | --- | --- | --- |
|  |  |  | | **Hazard ratio (95% CI)** | **P–value** | **Hazard ratio  (95% CI)** | **P–value** |
| Risk score |  | Within medicine | | 2.98 (2.55 – 3.48) | <0.001 | 2.45 (2.06 – 2.93) | <0.001 |
|  |  | Within critical care | | 1.77 (1.51 – 2.08) | <0.001 | 1.66 (1.38 – 2.02) | <0.001 |
|  |  | Within surgery | | 2.75 (2.10 – 3.61) | <0.001 | 2.36 (1.76 – 3.18) | <0.001 |
| Organism^a^ | *Non-ESBL E. coli* |  |  | *Reference* | <0.001 | *Reference* | 0.485 |
|  | ESBL producer |  |  | 1.79 (1.17 – 2.73) |  | 1.12 (0.69 – 1.81) |  |
|  | *Candida* |  |  | 2.56 (1.66 – 3.94) |  | 0.52 (0.28 – 0.97) |  |
|  | MRSA |  |  | 2.54 (1.62 – 4.00) |  | 1.34 (0.78 – 2.30) |  |
|  | MSSA |  |  | 1.74 (1.27 – 2.39) |  | 0.87 (0.61 – 1.26) |  |
|  | *P. aeruginosa* |  |  | 2.84 (2.01 – 4.00) |  | 0.80 (0.54 – 1.19) |  |
| Ward speciality ^b^ | *Medicine* |  |  | *Reference* | <0.001 | *Reference* | 0.026 |
|  | Critical care |  |  | 2.85 (2.24 – 3.63) |  | 1.82 (1.09 – 3.04) |  |
|  | Surgery |  |  | 0.54 (0.38 – 0.76) |  | 0.63 (0.39 – 1.03) |  |
| Central line present |  |  |  | 1.91 (1.41 – 2.57) | <0.001 | 0.87 (0.61 – 1.24) | 0.434 |
| Peripheral line present |  |  |  | 1.62 (1.14 – 2.31) | 0.008 | 0.89 (0.61 – 1.30) | 0.555 |
| Urinary catheter present |  |  |  | 2.67 (2.09 – 3.41) | <0.001 | 1.27 (0.95 – 1.71) | 0.106 |
| Average number of staff across the 3 shifts, per 10 beds |  | Within medicine | | 1.08 (0.95 – 1.23) | 0.216 | 1.09 (0.95 – 1.26) | 0.230 |
|  |  | Within critical care | | 1.02 (0.98 – 1.06) | 0.431 | 1.02 (0.98 – 1.06) | 0.373 |
|  |  | Within surgery | | 1.12 (0.76 – 1.67) | 0.560 | 1.11 (0.73 – 1.69) | 0.640 |
| Ward activity (number of admissions and discharges) per 10 beds |  | Within medicine | | 1.03 (1.01 – 1.06) | 0.002 | 1.03 (1.00 – 1.06) | 0.041 |
|  |  | Within critical care | | 1.07 (1.02 – 1.13) | 0.011 | 1.09 (1.01 – 1.17) | 0.024 |
|  |  | Within surgery | | 0.95 (0.84 – 1.07) | 0.397 | 0.97 (0.84 – 1.13) | 0.731 |
| Movement within ward speciality | One or more vs none |  |  | 0.63 (0.46 – 0.87) | 0.005 | 0.67 (0.46 – 0.96) | 0.028 |
| Movement to critical care | One or more vs none |  |  | 1.75 (1.19 – 2.57) | 0.004 | 1.31 (0.82 – 2.08) | 0.262 |
| Movement from critical care | One or more vs none |  |  | 0.48 (0.22 – 1.01) | 0.054 | 0.55 (0.25 – 1.24) | 0.152 |
| Movement from surgery to medicine | One or more vs none |  |  | 1.00 (0.41 – 2.43) | 0.996 | 1.56 (0.61 – 3.97) | 0.350 |
| Movement from medicine to surgery | One or more vs none |  |  | 0.48 (0.23 – 1.02) | 0.056 | 1.18 (0.51 – 2.74) | 0.697 |
| Time to appropriate therapy (days) |  | <5 days | Within non-ESBL *E. coli* | 7.62 (5.94 – 9.78) | <0.001 | 5.37 (4.01 – 7.18) | <0.001 |
|  |  |  | Within ESBL producer | 7.03 (5.03 – 9.83) | <0.001 | 6.14 (4.35 – 8.67) | <0.001 |
|  |  |  | Within *Candida* | 3.92 (2.98 – 5.17) | <0.001 | 2.72 (2.05 – 3.62) | <0.001 |
|  |  |  | Within MRSA | 4.18 (3.14 – 5.56) | <0.001 | 4.22 (3.13 – 5.68) | <0.001 |
|  |  |  | Within MSSA | 4.56 (3.78 – 5.50) | <0.001 | 3.63 (3.00 – 4.39) | <0.001 |
|  |  |  | Within *P. aeruginosa* | 4.39 (3.57 – 5.40) | <0.001 | 3.17 (2.58 – 3.89) | <0.001 |
|  |  | 5-7 days | Within non-ESBL *E. coli* | 0.79 (0.61 – 1.02) | 0.068 | 0.73 (0.55 – 0.97) | 0.030 |
|  |  |  | Within ESBL producer | 1.01 (0.83 – 1.23) | 0.935 | 1.06 (0.86 – 1.29) | 0.605 |
|  |  |  | Within *Candida* | 0.95 (0.82 – 1.11) | 0.552 | 0.92 (0.78 – 1.08) | 0.316 |
|  |  |  | Within MRSA | 0.98 (0.83 – 1.17) | 0.847 | 0.91 (0.76 – 1.09) | 0.303 |
|  |  |  | Within MSSA | 0.81 (0.65 – 1.00) | 0.050 | 0.83 (0.65 – 1.06) | 0.139 |
|  |  |  | Within *P. aeruginosa* | 0.95 (0.82 – 1.10) | 0.517 | 0.93 (0.79 – 1.10) | 0.388 |

**Notes:**

^a^ Effect of organism is given for the time period 0 to 5 days and when time to appropriate therapy is 1 day (multivariable model)

^b^ Effect of ward speciality is given for the median number of staff per 10 beds, median ward activity and median risk score (multivariable model)

**Supplemental figure 4**  Sensitivity analysis of primary outcome: removal of "36 hour rule" in definition of time to appropriate therapy


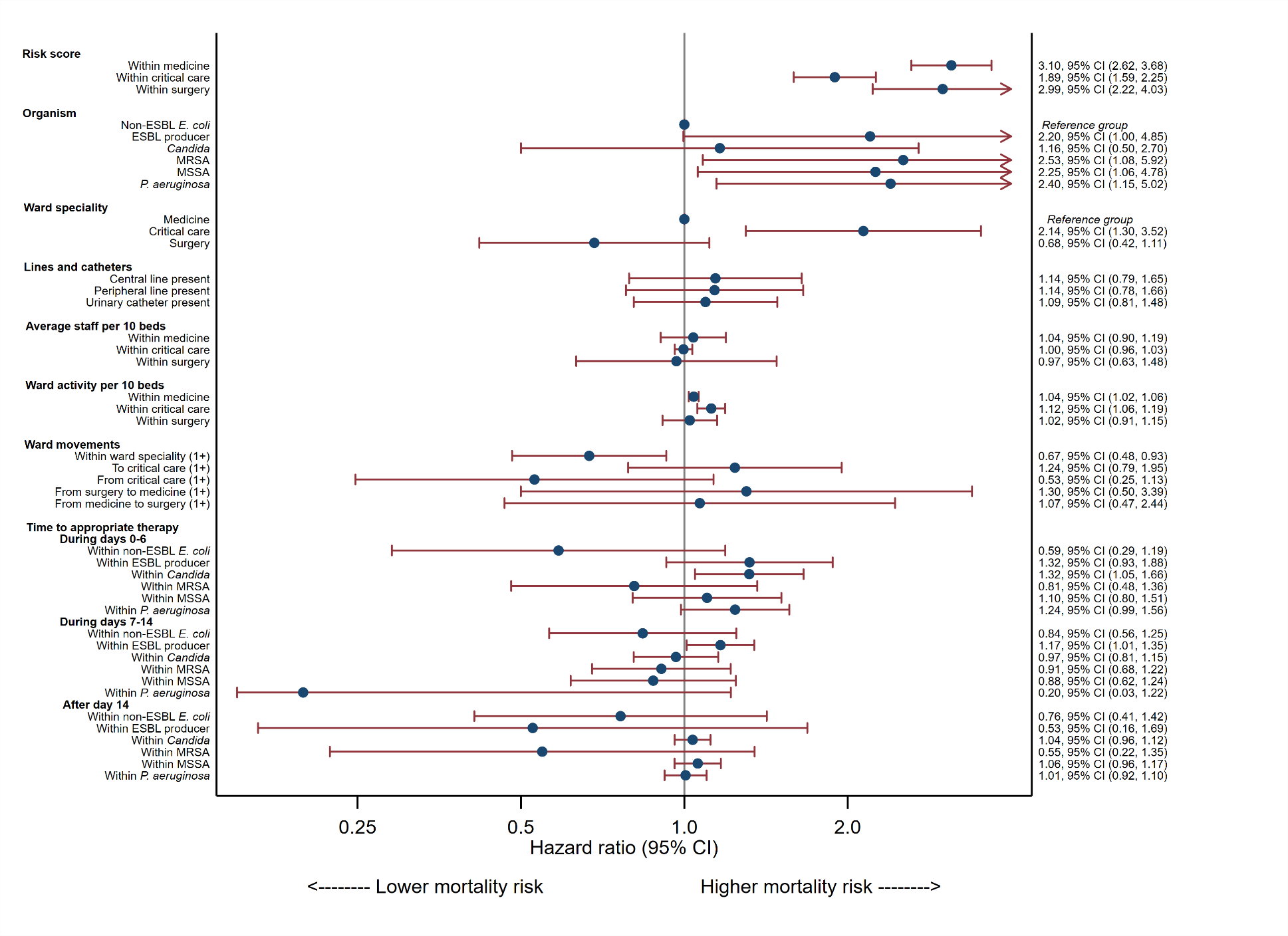


^a^ Effect of organism is given for the time period 0 to 6 days and when time to appropriate therapy is 1 day

^b^ Effect of ward speciality is given for the median number of staff per 10 beds, median ward activity and median risk score

***Abbreviations:*** *ESBL= Extended-spectrum beta-lactamase, MRSA- Methicillin-resistant S. aureus, MSSA= Methicillin-susceptible S. aureus, CI=Confidence interval*

**Supplemental figure 5**  Sensitivity analysis of primary outcome: 12-hour (in place of 36-hour) rule in definition of time to appropriate therapy


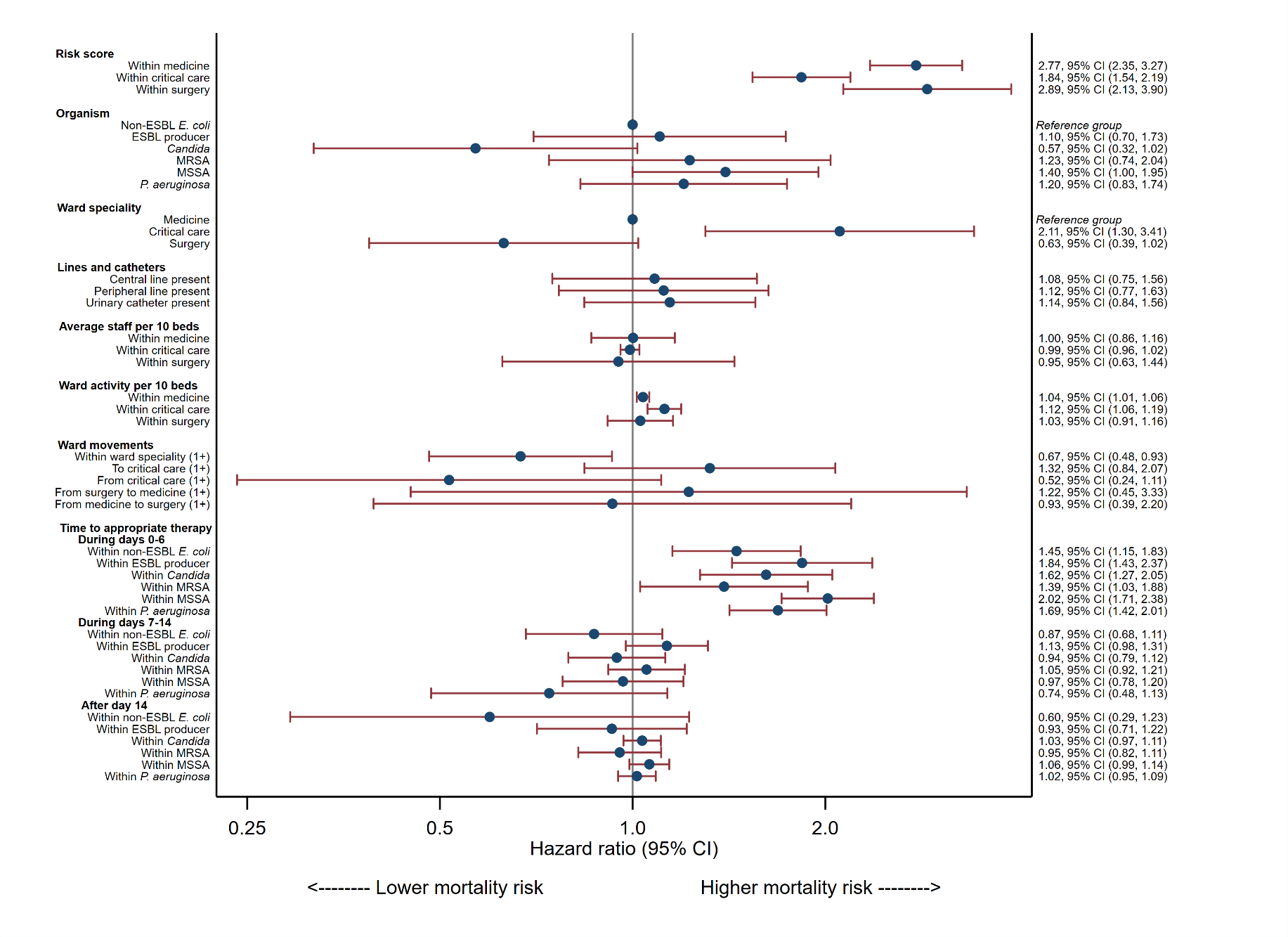


^a^ Effect of organism is given for the time period 0 to 6 days and when time to appropriate therapy is 1 day

^b^ Effect of ward speciality is given for the median number of staff per 10 beds, median ward activity and median risk score

***Abbreviations:*** *ESBL= Extended-spectrum beta-lactamase, MRSA- Methicillin-resistant S. aureus, MSSA= Methicillin-susceptible S. aureus, CI=Confidence interval*

**Supplemental figure 6** Sensitivity analysis of primary outcome: 24-hour (in place of 36-hour) rule in definition of time to appropriate therapy


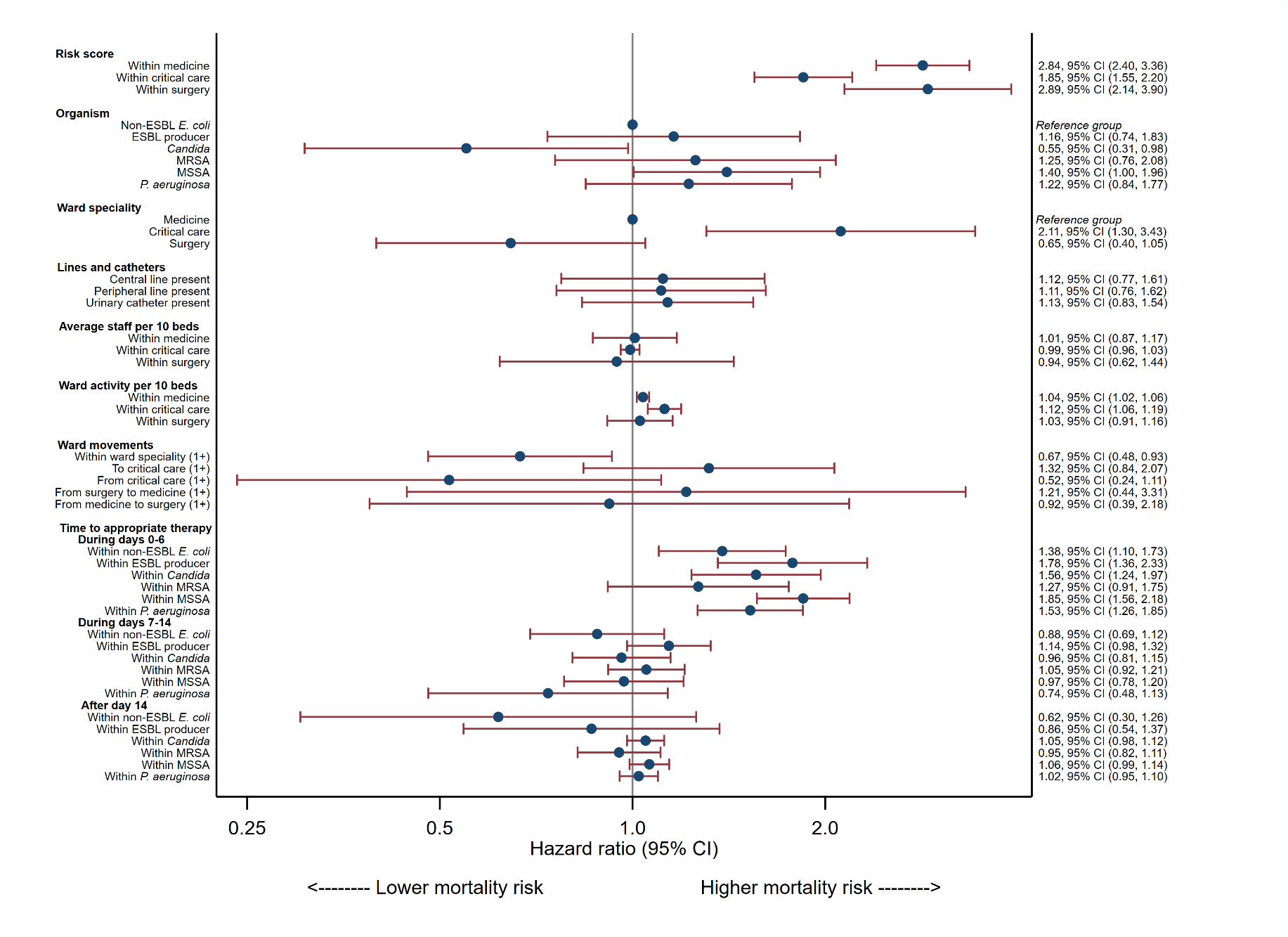


^a^ Effect of organism is given for the time period 0 to 6 days and when time to appropriate therapy is 1 day

^b^ Effect of ward speciality is given for the median number of staff per 10 beds, median ward activity and median risk score

***Abbreviations:*** *ESBL= Extended-spectrum beta-lactamase, MRSA- Methicillin-resistant S. aureus, MSSA= Methicillin-susceptible S. aureus, CI=Confidence interval*

**Supplemental figure 7** Sensitivity analysis of primary outcome: Complete case analysis
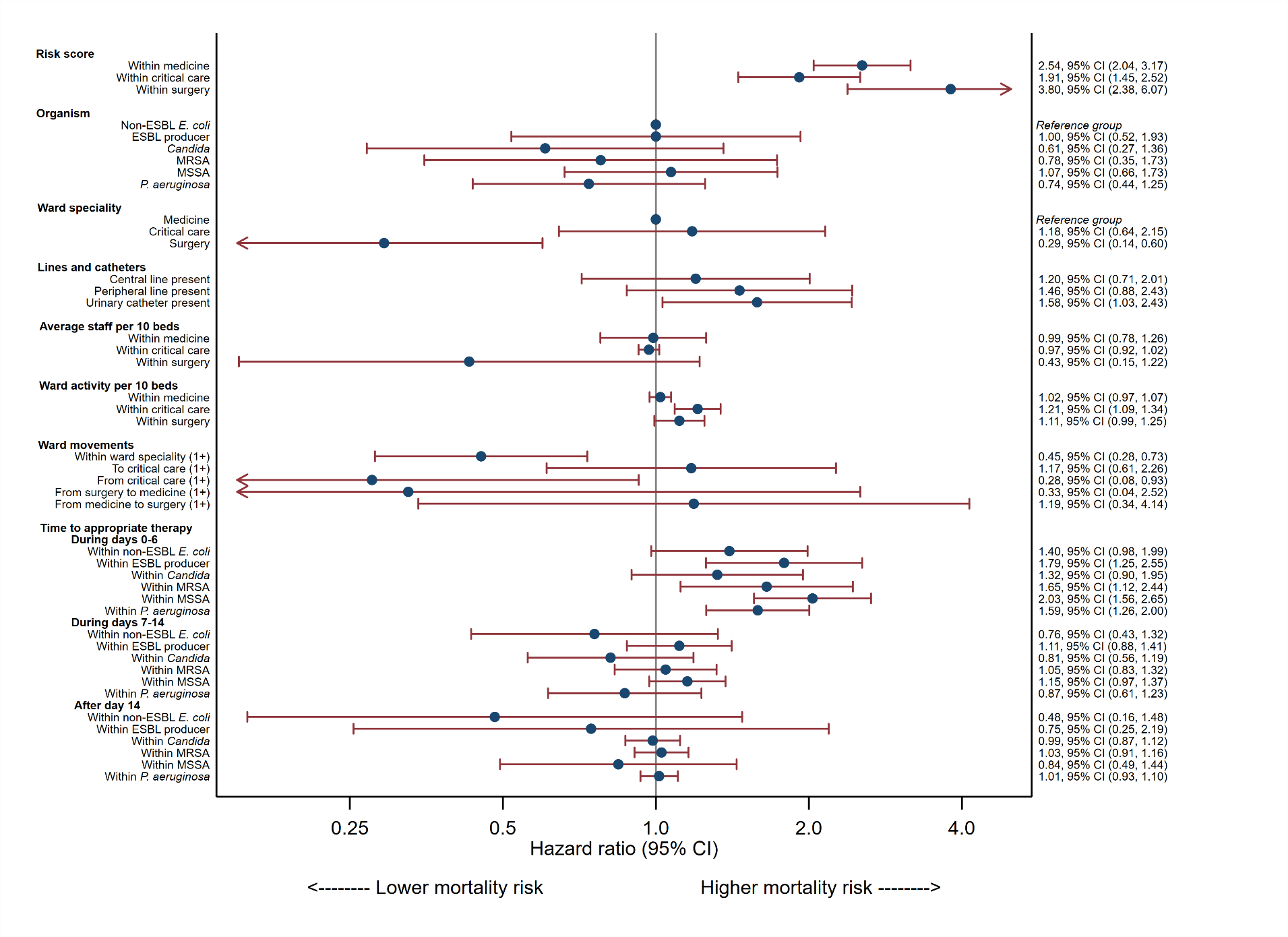


^a^ Effect of organism is given for the time period 0 to 6 days and when time to appropriate therapy is 1 day

^b^ Effect of ward speciality is given for the median number of staff per 10 beds, median ward activity and median risk score

***Abbreviations:*** *ESBL= Extended-spectrum beta-lactamase, MRSA- Methicillin-resistant S. aureus, MSSA= Methicillin-susceptible S. aureus, CI=Confidence interval*
